# Preclinical evidence of ginkgo biloba extract on diabetic nephropathy: a systematic review and meta-analysis

**DOI:** 10.3389/fphar.2025.1717777

**Published:** 2026-01-12

**Authors:** Jiangteng Liu, Ying Tang, Zhixun Guo, Zhichao Ruan, Yexin Chen, Yuanyuan Lin, Xingru Pan, Weijun Huang, Jinxi Zhao

**Affiliations:** 1 Dongzhimen Hospital, Beijing University of Chinese Medicine, Beijing, China; 2 Key Laboratory of Chinese Internal Medicine of Ministry of Education and Beijing, Beijing, China

**Keywords:** animal studies, diabetic nephropathy, Ginkgo biloba extract, meta-analysis, systematic review

## Abstract

**Background:**

Diabetic nephropathy (DN) is one of the common complications of diabetes, which is the leading cause of end-stage renal disease worldwide. Ginkgo biloba extract (GBE) has shown effectiveness in DN animal models and represents a promising therapeutic candidate. However, a comprehensive preclinical meta-analysis remains to be conducted, and the dose-time effect of GBE in the treatment of DN has not been evaluated.

**Objective:**

To evaluate the therapeutic effectiveness, mechanism and dose-time effect of GBE for DN by systematic review and meta-analysis.

**Methods:**

Seven databases (PubMed, Web of science, Embase, CBM, CNKI, Wanfang and VIP databases) were searched in this systematic review up to July 2025. Study quality was assessed using SYRCLE bias risk tool. STATA 14.0 software was employed to evaluate fasting blood glucose serum creatinine (SCr), blood urea nitrogen 24-h urine protein (24 h Upro), kidney index and indicators related to inflammatory response, oxidative stress, fibrosis, and glycolipid metabolism. The dose-time effect of Ginkgo biloba extract was evaluated by three-dimensional dose-time-effect analysis.

**Results:**

30 pertinent articles were included in the meta-analysis. Comparative analysis revealed that GBE exhibited statistically significant effect in reducing FBG, SCr, BUN, 24 h Upro, and KI. Furthermore, it also improved inflammatory indicators such as interleukin 1β (IL-1β), interleukin 6 (IL-6) and tumor necrosis factor α (TNF-α), as well as oxidative stress indicators like superoxide dismutase (SOD), malondialdehyde (MDA), antioxidation capability (AOC) and glutathione peroxidases (GSH-Px). Additionally, GBE showed positive effect in alleviating fibrosis and reducing serum total cholesterol (TC) and advanced glycation end products (AGEs). The dose-time-effect diagram showed that the rational dose of GBE for DN treatment was 36–200 mg/kg/d for 8–12 weeks.

**Conclusion:**

GBE may delay the progression of DN through multimodal mechanisms, including inhibition of inflammatory responses, attenuation of oxidative stress, suppression of fibrotic pathways, and modulation of glycolipid metabolism.

**Clinical Trial Registration:**

https://www.crd.york.ac.uk/PROSPERO/view/CRD420250652386.

## Introduction

Diabetes mellitus (DM), a chronic metabolic disorder defined by persistent hyperglycemia, predisposes individuals to multisystem complications (Niu et al., 2024). Diabetic nephropathy (DN) is one of the principal complications of DM and constitutes the leading etiology of end-stage renal disease (ESRD) (de Boer et al., 2022). About 30%–40% of DM patients develop DN, which will significantly increase the mortality rate of DM patients (Bonner et al., 2020; Thipsawat, 2021). The pathophysiology of DN involves multifactorial interactions, including metabolic abnormalities (chronic hyperglycemia and dyslipidemia), hemodynamic alterations, inflammatory responses and oxidative stress (Dwivedi and Sikarwar, 2025). Althoughcurrent therapeutic strategies employing sodium-glucose cotransporter 2 inhibitor (SGLT2i), glucagon-like peptide-1 receptor agonist (GLP-1RA) and selective non-steroidal glucocorticoid receptor antagonist (MRA) demonstrate partial efficacy in slowing DN progression (Epstein, 2015; Muskiet et al., 2015; Giglio et al., 2023), the progressive deterioration of renal function remains (Wheeler et al., 2021; Shetty et al., 2022; Giglio et al., 2023) and safer and more effective drugs are still urgently needed.

Natural botanical drugs show great potential in developing new therapeutic drugs for DN (Hu et al., 2023), including Ginkgo biloba extract (GBE), derived from the dried leaves of *ginkgo biloba L*. It has been widely used in the clinical management of heart disease, hyperlipidemia, diabetes and cerebrovascular disease (Achete de Souza et al., 2020; Hui et al., 2023). The standardized GBE, originally pioneered by Dr. Willmar Schwabe, contains various active metabolites (mainly total flavonol glycosides and terpene lactones) and has many pharmacological effects such as anti-inflammatory, anti-oxidation and anti-apoptosis (Xie et al., 2022). According to the *Pharmacopoeia of the People’s Republic of China*, on a dried basis, the content of total flavonol glycosides in GBE must not be less than 24.0%, and that of terpene lactones must not be less than 6.0%. As a botanical dietary supplement, GBE is recognized for its role in disease prevention and treatment and is welcomed in many countries (Trabert and Seifert, 2024).

In recent years, GBE has been found effective in treating DN via multiple pharmacological mechanisms, including inhibiting NLRP3 inflammasome activation (Li et al., 2019), increasing Nrf2-regulated HO-1 expression (Liu et al., 2024), and reducing ECM accumulation (Han et al., 2008). However, its precise mechanisms of action in treating DN remain unclear. Moreover, research in this field spans over three decades, leading to heterogeneity in outcome measures and postulated mechanistic pathways across studies. Currently, there is no meta-analysis based on preclinical studies to summarize the role of GBE in the treatment of DN. In this study, relevant preclinical studies were systematically reviewed, to comprehensively evaluate the protective effect and potential mechanism of GBE in DN models and support further high-quality research.

## Methods

This review adhered strictly to the PRISMA guidelines for systematic reviews and meta-analyses (Page et al., 2021) and was registered with PROSPERO (CRD420250652386).

### Search strategy

PubMed, Web of Science, Embase, CBM, CNKI, Wan Fang and VIP databases were searched for animal studies on treating DN with GBE. The search date was up to July 2025. Under the topic item, the search was performed in a combination of subject words and free words. These keywords were associated with DN and GBE, including “Diabetic Nephropathies,” “Nephropathies, Diabetic” and “Diabetic Kidney Disease,” as well as “Ginkgo biloba extract,” “Ginkgo leaf extract,” and “EGb 761.” The full search strategy has been presented in Supplementary Table S1.

### Eligibility criteria

Inclusion criteria: (1) Population: animal model of DN, including rats and mice; (2) Intervention: GBE, administration time and dose clear; (3) Comparison: untreated group or vehicle control; (4) Outcomes: FBG, SCr, BUN, 24 h Upro and KI, one or more of which must be covered. Other mechanism indicators, such as inflammation, oxidative stress, fibrosis indicators and so on.

Exclusion criteria: (1) Duplicated studies; (2) Review, network pharmacology, expert consensus, scientific and technological achievements; (3) Clinical research or *in vitro* experiments; (4) Treatment without GBE or combinations with other interventions; (5) Not DN animal model; (6) Full text not available; (7) Inconsistent outcome indicators or incomplete results.

### Data extraction

Two investigators independently assessed the included studies against predefined eligibility criteria, and extracted the following information: (1) The first author’s name and the publication year; (2) Species, sex, weight and number of animals in the experimental and model groups; (3) The establishment method of the model; (4) Characterisation of GBE: adherence to Consensus-based reporting guidelines for Phytochemical Characterisation of Medicinal Plant extracts (ConPhyMP) statement (Heinrich et al., 2022), batch number documentation required for commercial products; (5) The administration method, drug dose and duration; (6) Outcome indicators: The primary outcome indicators included FBG, SCr, BUN, 24 h Upro and KI. Other indicators included IL-1β, IL-6, TNF-α, SOD, MDA, GSH-Px, AOC, CAT, Col IV, MMP-2, TIMP-2, HbA1c, Insulin, TC, TG and AGEs.

All included data were presented as mean ± SD. The graphically represented data was obtained through WebPlotDigitizer 4.7 software. The SEM in the study was transformed into SD (SD = SEM × $\sqrt{n}$ ) (Sedgwick, 2015). If there were differences in the extraction of data, the two commentators reached a consensus through discussion, and those that remain unresolved were determined by the corresponding authors. When the study obtained the outcome indicators of multiple intervention durations, only the longest duration data were extracted. When the study contained multiple intervention doses, the mean and SD were combined using the formula (Sample size: N_1_ + N_2_, $\text{Mean}:\frac{N_{1}M_{1}+N_{2}M_{2}}{N_{1}+N_{2}}$ ; $\text{SD}:\sqrt{\frac{\left(N_{1}‐1\right)SD_{1}^{2}+\left(N_{2}‐1\right)SD_{2}^{2}+\frac{N_{1}N_{2}}{N_{1}+N_{2}}\left(M_{1}^{2}+M_{2}^{2}‐2M_{1}M_{2}\right)}{N_{1}+N_{2}‐1}}$ ).

### Risk bias evaluations

The methodological quality assessment was evaluated by the SYRCLE tool (Hooijmans et al., 2014). Risk bias evaluation was conducted by two independent reviewers. If they got different results, a consensus would be reached after discussion, or the third reviewer made the final decision. Quality assessment results were generated through RevMan 5.3 software. Results were shown in Supplementary Table S2.

### Statistical analysis

STATA 14.0 software was used for statistical analysis. Standardized Mean Difference (SMD) and 95% Confidence Interval (95% CI) were used to ascertain the summary statistics, with *p*-value < 0.05 (*p* < 0.05) signifying statistical significance. I-square (*I* ^*2*^) and P-heterogeneity were calculated to determine the heterogeneity and select the random effect model (*I* ^*2*^ > 50% or P < 0.1) or fixed effect model (*I* ^*2*^ ≤ 50% and P > 0.1). Moreover, causes of heterogeneity were identified through sensitivity analysis and subgroup analysis. Subgroup analysis was performed according to animal type (mice or rats), DN model (type 1 or type 2), administration method (gavage or intraperitoneal injection) and duration (duration < 8 weeks, 8 weeks ≤ duration < 12 weeks, duration ≥ 12 weeks). Potential publication bias was assessed through funnel plots and Egger’s regression test. If there was publication bias, pruning and filling methods were performed and the adjusted estimates were reported. The dose-time-effect relationship diagrams of FBG, SCr, BUN, 24 h Upro and KI were constructed to explore better treatment options.

## Results

### Study selection

**FIGURE 1:**
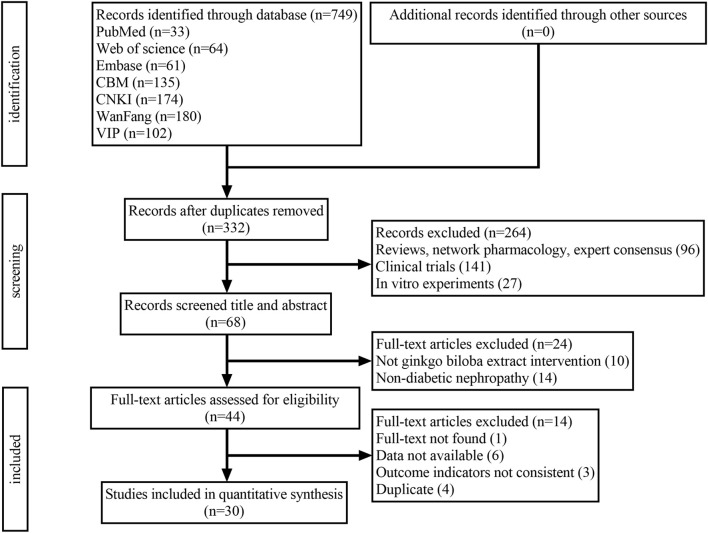
Flow chart of study selection.

According to the search strategy, 749 studies were retrieved from 7 databases. After combining all studies and removing duplicates, 332 studies were retained. Among these studies, 302 studies were excluded according to the exclusion criteria. Thirty eligible studies were ultimately retained in our meta-analysis (Figure 1).

### Study characteristics

30 studies contained 681 animals. Rats were used in 27 studies, including SD rats (14 studies) and Wistar rats (13 studies). Only three studies used mice, including C57BL/6N mice (1 study), KK/Ay mice (1 study) and DBA/2 mice (1 study). Regarding animal gender, 28 studies used male animals, one study used female animals, and one study used an equal number male and female animals. 27 studies described animal weights. In terms of modeling methods, 21 studies used STZ to establish an animal model, apart from spontaneous animal model used in one study. Seven studies used STZ in combination with either a high-fat diet or a high-fat, high-sugar diet, and one study opted for Alloxan. Regarding the type of DN, eight studies used a type 2 DN model, while 22 studies employed a type 1 DN model.

The GBE used in the studies were commercial products. Manufacturers were documented in 28 studies, with batch numbers specified in 17. For administration, 24 studies administered by oral gavage, and six studies administered by intraperitoneal injection. The shortest administration duration was 4 weeks, while the longest was 3 months. The dosage was from 5 to 300 mg/kg/d. Regarding the primary outcome indicators, 27 studies reported FBG, 18 studies reported SCr, 17 studies reported BUN, 14 studies reported 24 h Upro, and 16 studies reported KI. 22 studies reported the variations in markers associated with inflammation responses, oxidative stress, renal fibrosis, and glycolipid metabolism disorders (GLMDs) (Table 1). Relevant information on the GBE was shown in Supplementary Table S3.

**TABLE 1:** General characteristics of the articles.

Author, year	Species (sex, NE/NC)	Weight	Model	DN type	Dose	Administration	Duration	Outcomes
Chang et al. (2021)	DBA/2 mice (male, 10/5)	NR	STZ + diet	Ⅱ	50/200 (mg/kg/d)	Oral gavage	4 weeks	FBG, TC, TG
Chen (2006)	SD rats (male, 10/10)	200–300 g	STZ	Ⅰ	8 (mg/kg/d)	Intraperitoneal injection	5 weeks	FBG, SCr, 24 h Upro, KI
Chen et al. (2006)	SD rats (male, 10/9)	150–250 g	STZ + diet	Ⅱ	8 (mg/kg/d)	Intraperitoneal injection	2 months	24 h Upro, KI
Chen et al. (2024)	KK/Ay mice (male, 12/6)	NR	Spont	Ⅱ	100/200 (mg/kg/d)	Oral gavage	8 weeks	FBG, SCr, BUN, KI, AGEs
Du et al. (2019)	SD rats (male, 9/8)	180–220 g	STZ	Ⅰ	200 (mg/kg/d)	Oral gavage	10 weeks	FBG, SCr, BUN, 24 h Upro, KI
Han et al. (2008)	SD rats (male, 8/8)	210–240 g	STZ	Ⅰ	8 (mg/kg/d)	Intraperitoneal injection	7 weeks	FBG, FINS, TC, TG
Hou et al. (2005)	SD rats (male, 8/8)	180–200 g	STZ	Ⅰ	6.2 (mg/kg/d)	Oral gavage	10 weeks	FBG
Jiang et al. (2022)	SD rats (male, 12/12)	200–250 g	STZ	Ⅰ	96 (mg/kg/d)	Oral gavage	12 weeks	SCr, BUN, 24 h Upro, IL-6, TNF-α
Jing et al. (2005)	Wistar rats (male, 6/6)	200–260 g	STZ	Ⅰ	96 (mg/kg/d)	Oral gavage	12 weeks	FBG, IL-6, TNF-α
Li et al. (2011)	Wistar rats (male, 6/5)	200–230 g	STZ	Ⅰ	300 (mg/kg/d)	Oral gavage	20 weeks	FBG, SCr, 24 h Upro, MDA, AGEs
Li et al. (2019)	Wistar rats (male, 10/10)	200–250 g	STZ	Ⅰ	96 (mg/kg/d)	Oral gavage	12 weeks	FBG, SCr, BUN, KI, IL-6, Col IV, AGEs
Liao et al. (2000)	Wistar rats (male, 9/9)	150–200 g	Alloxan	Ⅰ	120 (mg/kg/d)	Oral gavage	8 weeks	FBG, SCr, BUN, 24 h Upro, KI, SOD, GSH-Px
Liu et al. (2008)	SD rats (male, 14/13)	180 ± 20 g	STZ	Ⅰ	100 (mg/kg/d)	Oral gavage	12 weeks	FBG, SCr, BUN, KI, CAT, SOD, GSH-Px, AOC
Liu et al. (2015)	Wistar rats (female, 8/7)	200–250 g	STZ + diet	Ⅱ	96 (mg/kg/d)	Oral gavage	12 weeks	FBG, SCr, BUN, 24 h Upro, IL-6, SOD, TC, TG, FINS
Liu et al. (2024)	Wistar rats (male, 30/10)	220–230 g	STZ + diet	Ⅱ	50/80/100 (mg/kg/d)	Oral gavage	12 weeks	SCr, BUN, IL-1β, IL-6, SOD, MDA
Lu (2007)	SD rats (male, 40/13)	150–190 g	STZ	Ⅰ	50/100/200 (mg/kg/d)	Oral gavage	12 weeks	FBG, SCr, BUN, 24 h Upro, KI, SOD, AOC, CAT, GSH-Px, MMP-2, TIMP-2, AGEs, HbA1c
Lu et al. (2015)	Wistar rats (male, 18/6)	180–220 g	STZ	Ⅰ	50/100/200 (mg/kg/d)	Oral gavage	8 weeks	FBG, SCr, BUN, 24 h Upro, KI, Col IV
Mao et al. (2008)	SD rats (male, 10/10)	180–220 g	STZ	Ⅰ	8 (mg/kg/d)	Oral gavage	8 weeks	FBG, SCr, BUN, KI, MMP-2, TIMP-2, TC, TG, HbA1c
Mu (2014)	Wistar rats (male, 20/10)	200 ± 20 g	STZ	Ⅰ	50/100 (mg/kg/d)	Oral gavage	3 months	FBG, SCr, BUN, 24 h Upro
Nian (2003)	Wistar rats (half male and half female, 10/10)	200–250 g	STZ + diet	Ⅱ	120 (mg/kg/d)	Oral gavage	8 weeks	FBG, BUN, SOD, MDA, TC, TG, HbA1c
Pang et al. (2020)	C57BL/6N mice (male, 10/10)	20 ± 3 g	STZ + diet	Ⅱ	36 (mg/kg/d)	Oral gavage	8 weeks	FBG, SCr, BUN, 24 h Upro, KI
Su (2013)	Wistar rats (male, 10/10)	200–220 g	STZ	Ⅰ	100 (mg/kg/d)	Oral gavage	12 weeks	FBG
Tang et al. (2011)	SD rats (male, 7/6)	180–220 g	STZ	Ⅰ	200 (mg/kg/d)	Oral gavage	6 weeks	FBG, 24 h Upro, KI
Wang et al. (2011)	SD rats (male, 20/20)	180–250 g	STZ	Ⅰ	8 (mg/kg/d)	Intraperitoneal injection	5 weeks	FBG, SCr, BUN, KI, MMP-2, TC, TG
Yang and Wang (2011)	SD rats (male, 6/6)	250–300 g	STZ	Ⅰ	200 (mg/kg/d)	Oral gavage	8 weeks	FBG, KI, MMP-2, Col IV
Yin et al. (2003)	SD rats (male, 17/8)	160–180 g	STZ	Ⅰ	75/150 (mg/kg/d)	Oral gavage	4 weeks	FBG, BUN, KI, AOC, HbA1c
Zhang et al. (2008)	Wistar rats (male, 8/8)	NR	STZ + diet	Ⅱ	8 (mg/kg/d)	Intraperitoneal injection	10 weeks	FBG, SOD, MDA
Zhang et al. (2017)	Wistar rats (male, 24/24)	200–250 g	STZ	Ⅰ	96 (mg/kg/d)	Oral gavage	12 weeks	FBG, IL-1β, TNF-α
Zhang et al. (2018)	Wistar rats (male, 10/10)	200–250 g	STZ	Ⅰ	96 (mg/kg/d)	Oral gavage	12 weeks	FBG, SCr, BUN, 24 h Upro, IL-6, TNF-α
Zheng (2011)	SD rats (male, 24/8)	200–220 g	STZ	Ⅰ	5/10/15 (mg/kg/d)	Intraperitoneal injection	8 weeks	FBG, SCr, BUN, 24 h Upro, MDA, SOD

### Quality assessment

**FIGURE 2:**
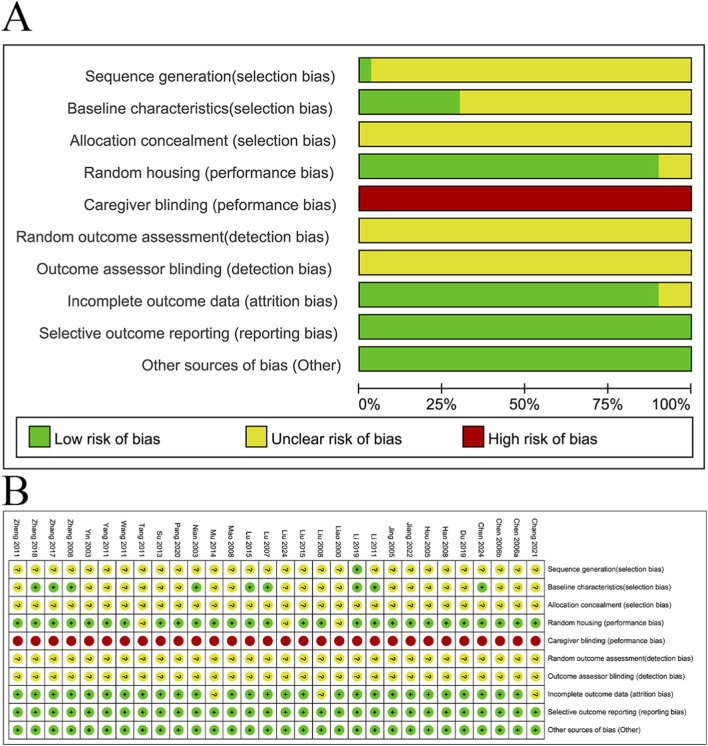
Deviation chart **(A)** and bias summary **(B)** incorporating research risks.

The quality of 30 included studies was evaluated by the SYRCLE tool and displayed via RevMan 5.3 software. Results showed that selective outcome reporting and other sources of bias were low risk, while caregiver blinding was high risk. Bias of allocation popularity, random outcome assessment, outcome assessor blinding were unknown risks. Among the included studies, 27 studies reported random housing, one study reported sequence generation, 9 studies reported baseline characteristics, and one studies had incomplete outcome data (Figure 2).

### Effectiveness

#### Primary outcomes

##### FBG

**FIGURE 3:**
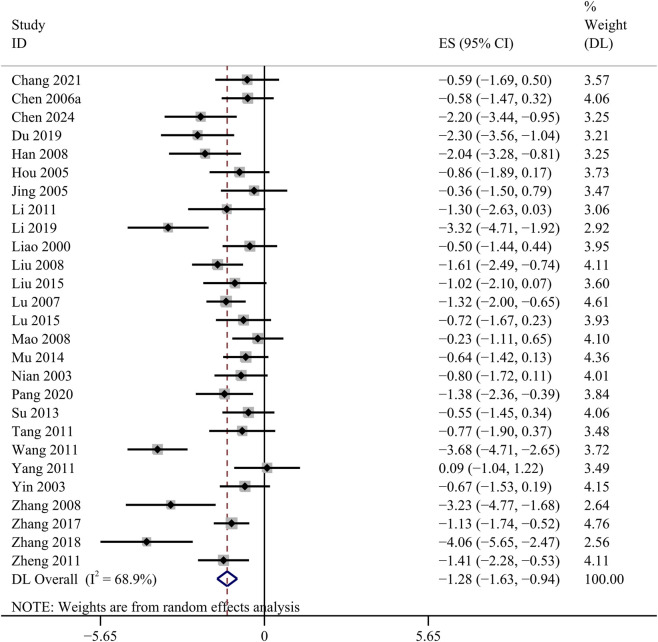
Forest plot of the effect of GBE on FBG.

FBG data were recorded in 27 studies. Comparative analysis revealed a statistically significant reduction in FBG in the GBE intervention group [SMD: −1.28, 95% CI (−1.63, −0.94), *p* = 0.000; *I* ^*2*^ = 68.9%, *p* = 0.000; Figure 3].

##### SCr

**FIGURE 4:**
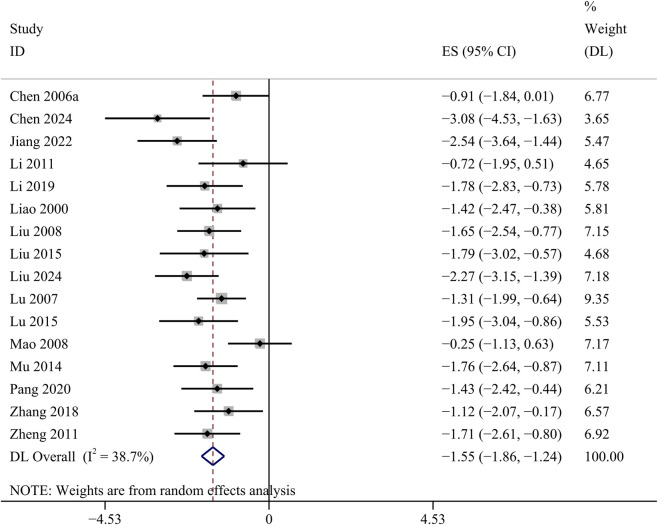
Forest plot of the effect of GBE on SCr.

18 studies provided changes in SCr. Among these studies, 2 studies had a substantial influence on the result, and only 16 studies were analyzed. The GBE demonstrated a statistically significant decrease in SCr concentrations [SMD: −1.55, 95% CI (−1.86, −1.24), *p* = 0.000; *I* ^*2*^ = 38.7%, *p* = 0.057; Figure 4].

##### BUN

**FIGURE 5:**
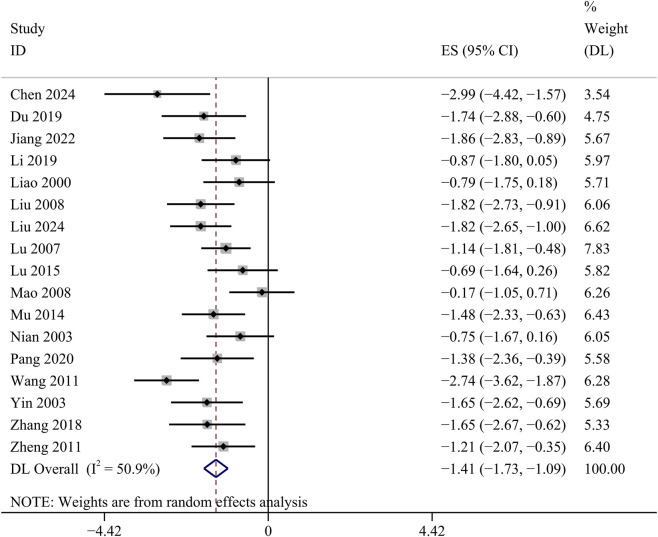
Forest plot of the effect of GBE on BUN.

17 studies detected BUN concentrations. Comparative analysis showed a statistically significant decrease in BUN concentrations in the GBE intervention group [SMD: −1.41, 95% CI (−1.73, −1.09), *p* = 0.000; *I* ^*2*^ = 50.9%, *p* = 0.008; Figure 5].

##### 24 h Upro

**FIGURE 6:**
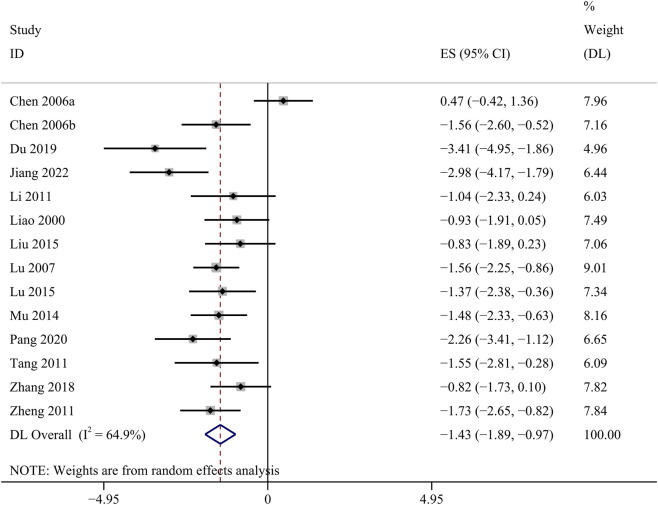
Forest plot of the effect of GBE on 24 h Upro.

14 studies investigated the changes of 24 h Upro. The GBE suggested a statistically significant reduction in 24 h Upro [SMD: −1.43, 95% CI (−1.89, −0.97), *p* = 0.000; *I* ^*2*^ = 64.9%, *p* = 0.000; Figure 6].

##### KI

**FIGURE 7:**
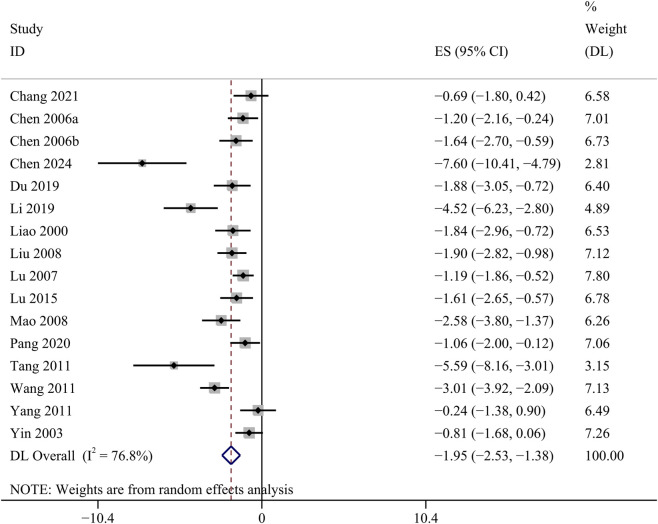
Forest plot of the effect of GBE on KI.

KI data were available in 16 studies. Comparative analysis demonstrated that GBE exhibited a statistically significant attenuation of KI [SMD: −1.95, 95% CI (−2.53, −1.38), *p* = 0.000; *I* ^*2*^ = 76.8%, *p* = 0.000; Figure 7].

#### Secondary outcomes

##### Inflammatory index

**FIGURE 8:**
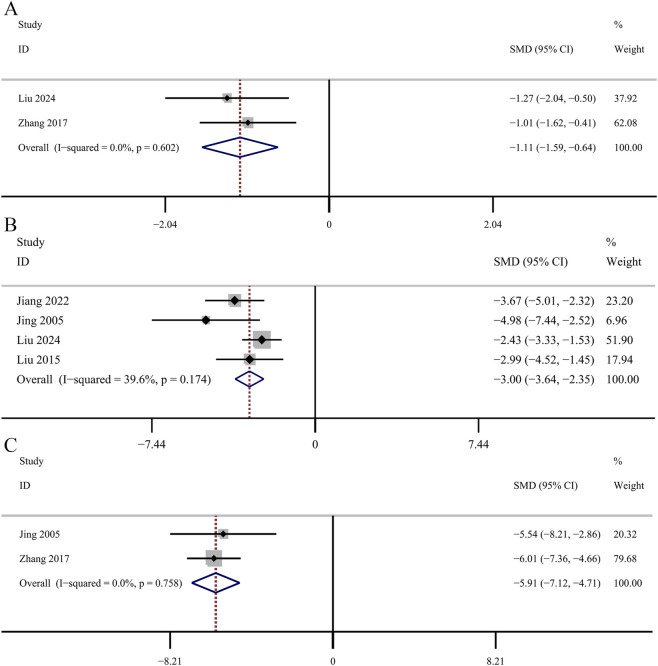
Forest plot of the effect of GBE on **(A)** IL-1β, **(B)** IL-6, **(C)** TNF-α.

Two studies investigated the changes of serum IL-1β. The reslut revealed that GBE exhibited statistically significant decrease in serum IL-1β concentrations [SMD: −1.11, 95% CI (−1.59, −0.64), *p* = 0.000; *I* ^*2*^ = 0.0%, *p* = 0.602; Figure 8A]. Four studies provided data on serum IL-6 concentrations. The GBE demonstrated a statistically significant decrease in serum IL-6 concentrations [SMD: −3.00, 95% CI (−3.64, −2.35), *p* = 0.000; *I* ^*2*^ = 39.6%, *p* = 0.174; Figure 8B]. Three studies used serum TNF-α as a research indicator. The data of one study had a great impact on the results and was excluded from our study. Comparative analysis demonstrated a statistically significant reduction in serum TNF-α concentrations in the GBE intervention group [SMD: −5.91, 95% CI (−7.12, −4.71), *p* = 0.000; *I* ^*2*^ = 0.0%, *p* = 0.758; Figure 8C].

##### Oxidative stress related indicators

**FIGURE 9:**
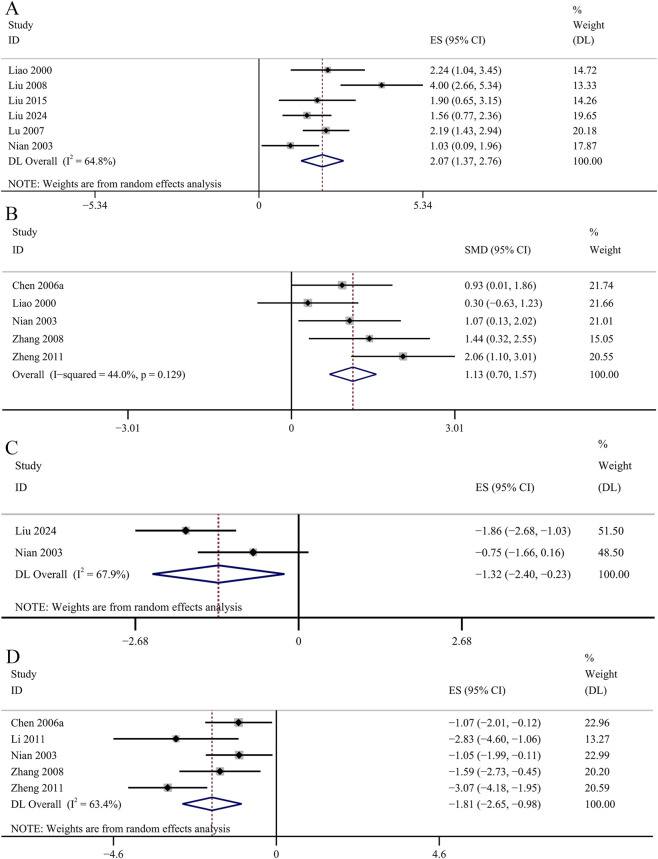
Forest plot of the effect of GBE on **(A)** serum SOD, **(B)** SOD in renal cortex, **(C)** serum MDA, **(D)** MDA in renal cortex.

**FIGURE 10:**
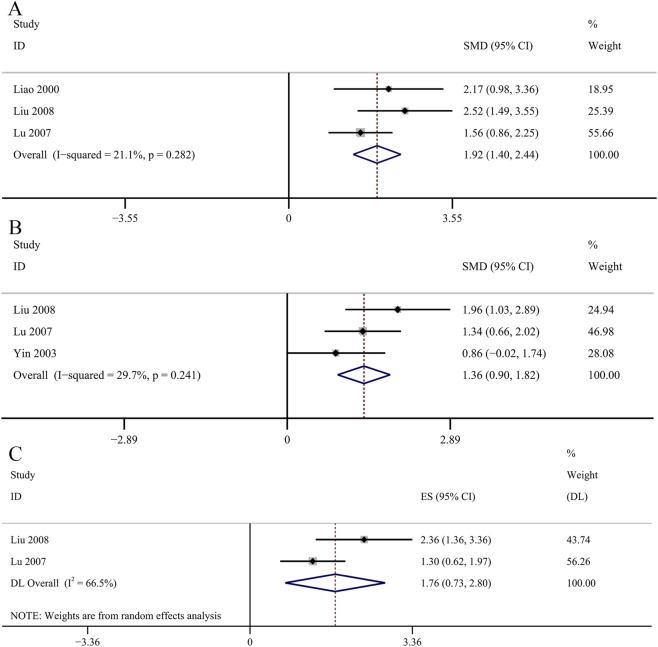
Forest plot of the effect of GBE on **(A)** GSH-Px, **(B)** AOC, **(C)** CAT.

The result of the six studies showed that GBE exhibited statistically significant increase in serum SOD concentrations [SMD: 2.07, 95% CI (1.37, 2.76), *p* = 0.003; *I* ^*2*^ = 64.8%, *p* = 0.014; Figure 9A]. The result of five studies suggested that GBE significantly increased the concentrations of SOD in renal cortex [SMD: 1.13, 95% CI (0.70, 1.57), *p* = 0.000; *I* ^*2*^ = 44%, *p* = 0.129; Figure 9B]. Two studies recorded the changes of serum MDA concentrations. No significant changes were observed in the GBE intervention group on serum MDA concentrations [SMD: −1.32, 95% CI (−2.40, −0.23), *p* = 0.253; *I* ^*2*^ = 67.9%, *p* = 0.077; Figure 9C]. Five studies recorded the efficacy of GBE in reducing MDA concentrations in renal cortex. Comparative analysis revealed that GBE exhibited statistically significant reduction in the concentrations of MDA in renal cortex [SMD: −1.81, 95% CI (−2.65, −0.98), *p* = 0.013; *I* ^*2*^ = 63.4%, *p* = 0.027; Figure 9D]. Three studies evaluated serum GSH-Px concentrations. The GBE demonstrated a statistically significant increase in serum GSH-Px concentrations [SMD: 1.92, 95% CI (1.40, 2.44), *p* = 0.000; *I* ^*2*^ = 21.1%, *p* = 0.282; Figure 10A]. Serum AOC was used as an outcome indicator in three studies. The result demonstrated a statistically significant increase in serum AOC concentrations in the GBE intervention group [SMD: 1.36, 95% CI (0.90, 1.82), *p* = 0.000; *I* ^*2*^ = 29.7%, *p* = 0.241; Figure 10B]. Two studies evaluated serum CAT concentrations. No significant changes were observed in the GBE intervention group on serum CAT concentrations [SMD: 1.76, 95% CI (0.73, 2.80), *p* = 0.185; *I* ^*2*^ = 66.5%, *p* = 0.084; Figure 10C].

##### Effect on fibrosis

**FIGURE 11:**
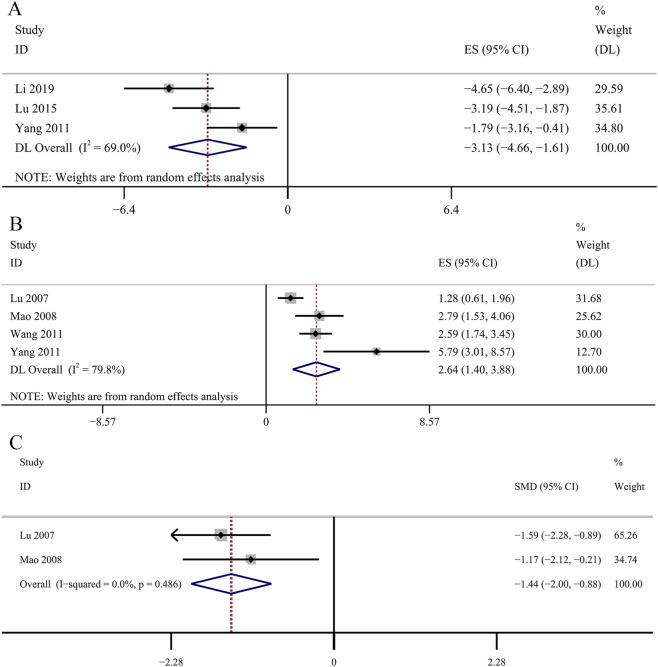
Forest plot of the effect of GBE on **(A)** Col IV, **(B)** MMP-2, **(C)** TIMP-2.

Three studies reported the determination of Col IV. Comparative analysis revealed no statistically significant impact of the GBE intervention group on Col IV concentrations [SMD: −3.13, 95% CI (−4.66, −1.61), *p* = 0.061; *I* ^*2*^ = 69.0%, *p* = 0.04; Figure 11A]. The result of the four studies demonstrated that GBE exhibited statistically significant increase in the concentrations of MMP-2 [SMD: 2.64, 95% CI (1.40, 3.88), *p* = 0.045; *I* ^*2*^ = 79.8%, *p* = 0.002; Figure 11B]. Three studies evaluated TIMP-2 concentrations. The result suggested a statistically significant reduction in TIMP-2 concentrations in the GBE intervention group [SMD: −1.44, 95% CI (−2.00, −0.88), *p* = 0.000; *I* ^*2*^ = 0.0%, *p* = 0.486; Figure 11C].

##### Regulation of glycolipid metabolism

**FIGURE 12:**
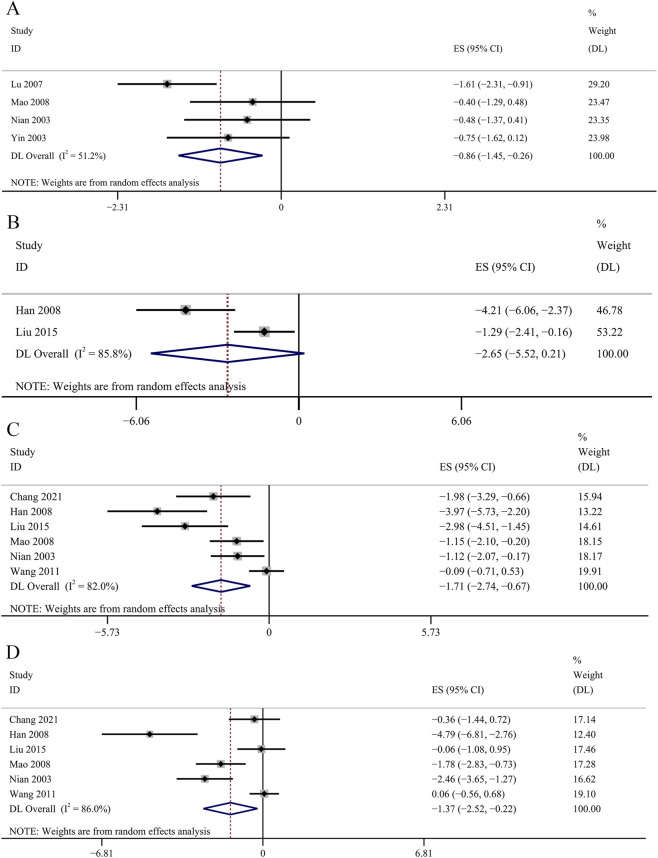
Forest plot of the effect of GBE on **(A)** HbA1c, **(B)** Insulin, **(C)** TC, **(D)** TG.

**FIGURE 13:**
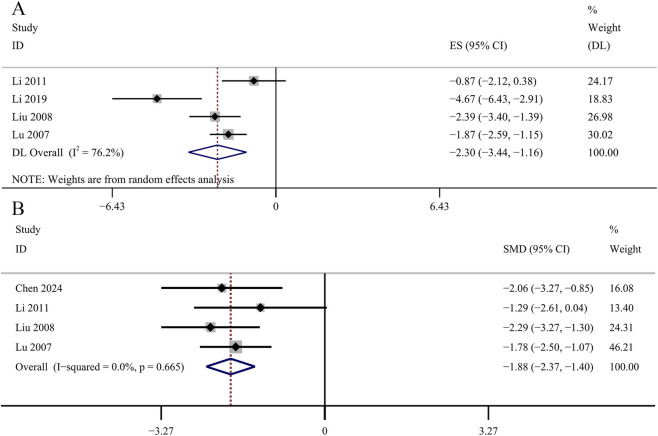
Forest plot of the effect of GBE on **(A)** AGEs in renal cortex and **(B)** serum AGEs.

HbA1c was used as an outcome indicator in four studies. No significant changes were observed in the GBE intervention group on HbA1c levels [SMD: −0.86, 95% CI (−1.45, −0.26), *p* = 0.066; *I* ^*2*^ = 51.2%, *p* = 0.105; Figure 12A]. Two studies evaluated insulin concentrations. The meta-analysis revealed no statistically significant impact of the GBE intervention group on insulin concentrations [SMD: −2.65, 95% CI (−5.52, 0.21), *p* = 0.320; *I* ^*2*^ = 85.8%, *p* = 0.008; Figure 12B]. Six studies recorded the changes of TC. The result revealed that GBE exhibited statistically significant reduction in TC concentrations [SMD: −1.71, 95% CI (−2.74, −0.67), *p* = 0.028; *I* ^*2*^ = 82.0%, *p* = 0.000; Figure 12C]. The result of 6 studies suggesed that GBE demonstrated no statistically significant impact on TG concentrations [SMD: −1.37, 95% CI (−2.52, −0.22), *p* = 0.108; *I* ^*2*^ = 86.0%, *p* = 0.000; Figure 12D]. Four studies reported the determination of AGEs in renal cortex. No significant changes were observed in the GBE intervention group on AGEs in renal cortex [SMD: −2.30, 95% CI (−3.44, −1.16), *p* = 0.054; *I* ^*2*^ = 76.2%, *p* = 0.006; Figure 13A]. Four studies recorded serum AGEs level. The meta-analysis indicated a statistically significant decrease in serum AGEs concentrations following GBE intervention [SMD: −1.88, 95% CI (−2.37, −1.40), *p* = 0.000; *I* ^*2*^ = 0.0%, *p* = 0.665; Figure 13B].

##### Sensitivity analysis

Sensitivity analysis was conducted based on the primary outcomes included in the meta-analysis. The result showed that no significant change was observed when each study was excluded from the comprehensive analysis in turn (Supplementary Figure S1).

##### Subgroup analysis

Subgroup analysis of FBG, SCr, BUN, 24 h Upro and KI was performed according to animal species, DN model, administration methods and duration.

For FBG, subgroup analysis by animal species revealed that heterogeneity was lower in mice studies, which also demonstrated the largest effect size [SMD: −1.35, 95% CI (−2.21, −0.50), *p* = 0.002; *I* ^*2*^ = 44.6%, *p* = 0.165].

For SCr, subgroup analysis by DN model type showed reduced heterogeneity in both subgroups, and type 2 DN demonstrated the greatest effect size [SMD: −2.06, 95% CI (−2.68, −1.44), *p* = 0.000; *I* ^*2*^ = 22.2%, *p* = 0.278]. Subgroup analysis by administration methods indicated that oral gavage yielded a smaller effect size than intraperitoneal injection [SMD: −1.31, 95% CI (−2.09, −0.53), *p* = 0.001; *I* ^*2*^ = 30.6%, *p* = 0.230], but heterogeneity was reduced in this subgroup analysis [SMD: −1.59, 95% CI (−1.94, −1.24), *p* = 0.000; *I* ^*2*^ = 42.4%, *p* = 0.047]. In the subgroup analysis based on duration, the long-duration subgroup exhibited markedly reduced heterogeneity and the largest effect size [SMD: −1.65, 95% CI (−1.99, −1.32), *p* = 0.000; *I* ^*2*^ = 10.9%, *p* = 0.344].

For BUN, the subgroup analysis by duration showed that the long-duration subgroup had significantly lower heterogeneity and the most pronounced effect size [SMD: −1.48, 95% CI (−1.80, −1.16), *p* = 0.000; *I* ^*2*^ = 0.0%, *p* = 0.603].

For 24 h UPro, subgroup analysis by DN model type revealed that the type 2 DN subgroup had reduced heterogeneity and demonstrated the greatest effect size [SMD: −1.53, 95% CI (−2.33, −0.73), *p* = 0.000; *I* ^*2*^ = 38.2%, *p* = 0.198]. In the analysis stratified by duration, the medium-duration subgroup showed a significant reduction in heterogeneity and the largest effect size [SMD: −1.74, 95% CI (−2.31, −1.17), p = 0.000; I2 = 40.8%, p = 0.133].

Other subgroup analyses did not show significant changes in heterogeneity. The results are shown in Supplementary Table S4.

##### Publication bias

**FIGURE 14:**
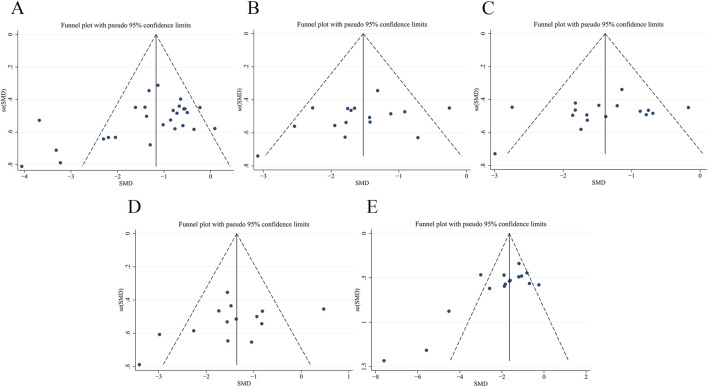
Publication bias of funnel plots of **(A)** FBG, **(B)** SCr, **(C)** BUN, **(D)** 24 h Upro, **(E)** KI.

Bias analysis was performed on FBG (Figure 14A), SCr (Figure 14B), BUN (Figure 14C), 24 h Upro (Figure 14D) and KI (Figure 14E). The results of Egger’s test showed that the bias of FBG (*p* = 0.024) and KI (*p* = 0.003) was statistically significant. SCr (*p* = 0.183), BUN (*p* = 0.221) and 24 h Upro (*p* = 0.167) publication bias was not statistically significant. Trim-and-fill analysis indicated that funnel plot asymmetry for FBG and KI could be resolved by imputing two and one studies, respectively. The specific data are shown in Supplementary Figures S2, S3 and Supplementary Table S5.

##### Dose-time-effect analysis

**FIGURE 15:**
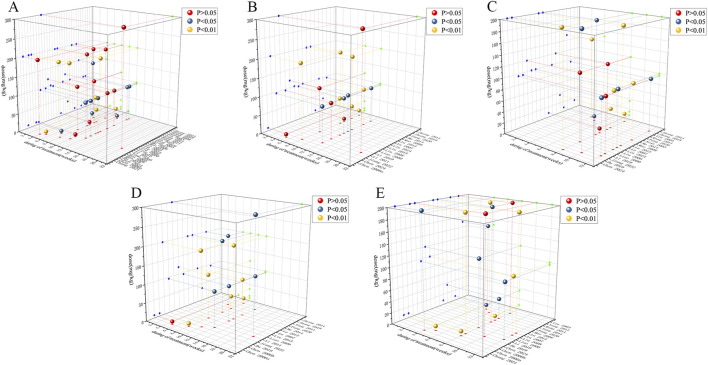
3D dose-time-effect images for **(A)** FBG, **(B)** SCr, **(C)** BUN, **(D)** 24 h Upro, **(E)** KI.

Three-dimensional image was constructed to study whether the dose and duration of administration have an effect on the therapeutic effect of GBE. Due to the limited number of studies administering GBE via intraperitoneal injection, the analysis was restricted to using oral administration. The effective dose of oral GBE to improve FBG (Figure 15A), BUN (Figure 15C) and KI (Figure 15E) was 36–200 mg/kg/d for 4–12 weeks, while to improve SCr, the duration should be 8–12 weeks (Figure 15B). The dose of oral GBE to reduce 24 h Upro was 36–300 mg/kg/d, and the duration was 6–12 weeks (Figure 15D). In general, GBE was administered at a dose of 36–200 mg/kg/d for 8–12 weeks, showing beneficial effects.

## Discussion

### Summary of evidence

A total of 30 studies were included in the meta-analysis. The included animal studies exhibited diversity in species selection, modeling methods, as well as the timing and dosage of administration. It should be noted that over 90% of the studies utilized male mice, which may bring some bias to this study. Furthermore, this practice obscures critical sex-based differences that could inform clinical research and may exacerbate the reproducibility crisis in preclinical biomedical research. At present, some scholars have advocated for the importance of gender inclusion in preclinical studies to ensure rigorous scientific protection. Regarding quality evaluation, it is recommended that researchers should describe the sequence generation, allocation concealment and baseline characteristics of animal experiments, report the research data completely, and minimize various biases.

The results demonstrated that GBE exerted significant protective efficacy in experimental DN models. The GBE demonstrated statistically significant attenuation of efficacy measures, including FBG, SCr, BUN, 24 h Upro and KI. After treatment with GBE, the concentrations of serum IL-1β, IL-6 and TNF-α were effectively reduced, indicating that GBE had the effect of reducing inflammation. In terms of oxidative stress indicators, GBE could increase serum SOD, GSH-Px, AOC and the concentrations of SOD in renal cortex, and reduce the concentrations of MDA in renal cortex. In terms of anti-fibrosis, the concentrations of MMP-2 in the GBE intervention group was increased and the concentrations of TIMP-2 was decreased, indicating that the fibrosis was improved. In addition, GBE also reduced serum TC and AGEs concentrations, and had the effect of improving glycolipid metabolism.

Notably, the meta-analysis did not reveal statistically significant effects of GBE on the levels of AGEs in the renal cortex, HbA1c and insulin. However, a narrative synthesis of four studies reporting on AGEs indicated that GBE significantly reduced renal cortical AGE levels. Because of the different animal strains, the inconsistent units of measurement results, and the small number of included literatures, no subgroup study was conducted, which may lead to a certain bias. Results showed that GBE did not significantly improve HbA1c, which may be related to the low dose of GBE and different animal strains. Although meta-analysis indicated that GBE significantly reduced insulin levels, the result is not reliable due to small sample size, variations in the animal strains and sex bias.

The results also showed that the analysis of the primary outcome indicators had great heterogeneity. Subgroup analysis showed that animal species may be the source of FBG heterogeneity, and DN model may be the source of SCr and 24 h Upro heterogeneity. For the specific administration of GBE, the mode of administration may be the source of SCr heterogeneity, and the duration may be the source of SCr, BUN and 24 h Upro heterogeneity. Nevertheless, the precise reasons for the heterogeneity remain incompletely understood. It is also important to note that, while commercially available GBE products comply with pharmacopeial standards, few studies have employed multiple analytical methods to comprehensively characterize their chemical composition. Variability in the chemical composition of GBE may constitute an important source of the observed heterogeneity. This highlights the necessity for standardized and transparent reporting in future animal and clinical studies.

In the primary outcome indicators, FBG and KI have publication bias. After the adjustment of the trim-and-fill analysis, the effect value of the index changed slightly, but it did not affect the statistical significance of the results, indicating that the overall results were robust. The dose-time-effect relationship was performed that GBE had a good therapeutic effect at a dose of 36–200 mg/kg/d for 8–12 weeks in all animal species and models.

This study comprehensively analyzed the effect of GBE in the treatment of DN, and provided evidence for further clinical research to promote the clinical application of GBE. It is important to acknowledge that the included studies often lack a comprehensive assessment of the specific molecular pathways through which GBE exerts its effects on DN. Therefore, further high-quality preclinical investigations are warranted to elucidate these mechanisms and strengthen the translational foundation.

### Possible protective mechanisms of GBE

#### Anti-inflammatory effects

Although DN was traditionally viewed as a metabolic disorder, growing evidence supports inflammation as a key driver (Chen J. H. et al., 2022). Under diabetic conditions, sustained hyperglycemia and oxidative stress collectively induce pathological activation of multiple signaling pathways. These metabolic disturbances trigger immune cell recruitment/activation and upregulate inflammatory gene expression, thereby promoting the release of proinflammatory cytokines including interleukin 1β (IL-1β) and tumor necrosis factor α (TNF-α). This cytokine-driven inflammatory cascade promotes chronic renal inflammation, ultimately resulting in structural remodeling and tubulointerstitial fibrosis development (Rayego-Mateos et al., 2020; Tang and Yiu, 2020; Chen J. H. et al., 2022). Preclinical studies have demonstrated that, GBE intervention significantly reduces renal expression of IL-1β in DN models (Chen et al., 2006; Liu et al., 2015; Zhang et al., 2017; 2018; Li et al., 2019; Jiang et al., 2022; Chen et al., 2024; Liu et al., 2024), indicating its anti-inflammatory potential through suppression of inflammatory mediator production. Activation of NOD-like receptor pyrin domain-containing protein 3 (NLRP3) inflammasome can amplify the inflammatory cascade and promote pyroptosis (Wan et al., 2022). Experimental evidence suggests GBE preserves renal function through NLRP3 inflammasome inhibition, reducing IL-1β/TNF-α synthesis and alleviating renal inflammation in DN rats (Liu et al., 2015; Zhang et al., 2017; 2018; Li et al., 2019). Toll-like receptor 4 (TLR4) is a canonical pattern recognition receptor (PRR) expressed on cell surfaces, which can initiate immune responses, subsequently activating intracellular signaling (Li W. J. et al., 2022). Nuclear factor κB (NF-κB) interacts with TLR4 to mediate NLRP3 inflammasome activation (Zhang et al., 2021; Li W. J. et al., 2022; Zhang et al., 2022). Jing et al. (2005) found that GBE inhibits NF-κB activation, consequently reducing TNF-α expression. Zhang’s research documented decreased renal TLR4 levels and corresponding reductions in TNF-α and IL-6 following GBE treatment, suggesting coordinated inhibition of the TLR4/NF-κB/NLRP3 signaling axis (Zhang et al., 2018). These collective findings establish that GBE exerts renoprotection primarily through multi-targeted suppression of proinflammatory factor synthesis and release in DN pathogenesis.

#### Anti-oxidative stress

Oxidative stress constitutes a pivotal mechanism driving DN pathogenesis (Charlton et al., 2020; Darenskaya et al., 2021; Iacobini et al., 2021). Under physiological conditions, reactive oxygen species (ROS) generated during cellular metabolism regulate essential processes including cell proliferation, differentiation, and signal transduction. Pathological conditions disrupt the ROS-antioxidant defense system (AOD) balance, resulting in oxidative stress through ROS overproduction with AOD impairment. This redox imbalance damages macromolecules (proteins, lipids, DNA), leading to inflammation responses, apoptotic signaling and cellular senescence (Darenskaya et al., 2023). Oxidative modification generates specific biomarkers: lipid peroxidation yields malondialdehyde (MDA), while protein oxidation will produce advanced glycation end products (AGEs). The AOD system neutralizes ROS mainly through reduced superoxide dismutase (SOD), glutathione peroxidases (GSH-Px), catalases (CAT) and so on (Darenskaya et al., 2023). Experimental evidence demonstrates that GBE effectively scavenges excessive ROS in dose-dependent manners *in vivo* and *in vitro* DN models confirming its antioxidant capacity (Li Y. et al., 2022). Numbers of studies document that GBE intervention elevates renal SOD and GSH-Px concentrations while reducing MDA in DN rats (Liao et al., 2000; Nian, 2003; Yin et al., 2003; Chen, 2006; Lu, 2007; Liu et al., 2008; Zhang et al., 2008; Li et al., 2011; Zheng, 2011; Liu et al., 2015; Chen J. et al., 2022; Liu et al., 2024), suggesting its renoprotection partially stems from antioxidant modulation. Nuclear factor erythroid 2 related factor 2 (Nrf2) is the main regulator of the endogenous antioxidant system, which can regulate the synthesis of various antioxidant enzymes (Li Y. et al., 2022). Chang et al. (2021) demonstrated that GBE treatment in DN mice, upregulated Nrf2-regulated antioxidant enzyme heme oxygenase 1 (HO-1) expression, attenuating oxidized low-density lipoprotein uptake and high glucose-induced ROS generation in podocytes while HO-1 inhibition abolished GBE’s podocyte-protective effects. Similarly, the study of Liu et al. (2024) revealed GBE administration in DN rats significantly increased Nrf2-regulated HO-1 and SOD levels. Given that these antioxidants effectors operate downstream of Nrf2/ARE signaling, current findings strongly implicate GBE’s therapeutic antioxidant effects in DN involve Nrf2/ARE pathway activation.

#### Anti-fibrosis effects

Growing evidence identifies fibrosis as a hallmark pathological feature in DN progression, driven primarily by excessive extracellular matrix (ECM) accumulation (Distler et al., 2019; Zeng et al., 2019; Han et al., 2021; Wang et al., 2023). These processes are modulated by TGF-β, AGEs, and TNF-α. Previous studies (Chen et al., 2006; Su, 2013; Lu et al., 2015; Li et al., 2019; Han et al., 2021) have shown that GBE significantly reduces high glucose-induced ECM protein deposition in both cellular and animal DN models. These studies confirm that GBE has a clear anti-fibrotic effect. The ECM degradation-regeneration balance is critically regulated by matrix metalloproteinases (MMPs) and tissue inhibitor of matrix metalloproteinases (TIMPs). The dysregulation of MMP/TIMP ratios, particularly MMP-2/MMP-9 suppression and TIMP-2 overexpression, constitutes a key driver of renal fibrosis (Narula et al., 2018; Tang et al., 2021). Substantial studies (Chen et al., 2006; Lu, 2007; Mao et al., 2008; Wang et al., 2011; Yang and Wang, 2011) indicate that GBE restores MMP-2/MMP-9 expression while inhibiting TIMP-2 in renal tissue, thereby promoting ECM degradation and mitigating fibrosis. TGF-β activates and promotes the activation and proliferation of myofibroblasts and induces epithelial-mesenchymal transition (EMT). As the major fibroblast cytokine, which regulate renal fibrosis through TGF-β/Smad pathway activation (Zeng et al., 2019). Han et al. (2008) reported that GBE downregulates the expression of TGF-β1 in DN rats, effectively reducing the accumulation of ECM and delaying the process of tubulointerstitial fibrosis. Qiu et al. (2023) found that overexpression of NQO1 attenuates high glucose-induced TGF-β1/Smad signaling pathways, thereby inhibiting proinflammatory cytokine release, ECM deposition and EMT progression. Combined with previous studies (Li Y. et al., 2022; Liu et al., 2024), GBE upregulates the expression of HO-1, NQO1 and SOD by activating the Nrf2/ARE signaling pathway, and exert anti-inflammatory and anti-fibrosis effects through NQO1-mediated mechanisms. Renal fibrosis remains a primary contributor to ESRD through complex interactions involving inflammation, oxidative stress, hypoxia (Wang et al., 2023). Therefore, the research on the mechanism of GBE anti-renal fibrosis needs to be further developed.

#### Regulation of glycolipid metabolism

GLMDs are characterized by dysregulated glucose/lipid synthesis, breakdown, and absorption within the body, including hyperglycemia, insulin resistance (IR) and dyslipidemia, which contribute significantly to DN pathogenesis (Iacobini et al., 2021; Chen et al., 2022c; de Lima et al., 2024). High glucose environment can damage various cells in the kidney, promote the polyol pathway, promote the formation of AGEs, induce metabolic inflammation, oxidative stress and excessive ECM formation, and ultimately lead to DN. Dyslipidemia is closely related to high glucose and IR. Excessive accumulation of lipids and lipid droplets in glomeruli and tubules will activate a variety of signaling pathways, leading to kidney damage (Wu et al., 2023). Extensive studies (Han et al., 2021; Hou et al., 2005; Lu et al., 2015; Mu, 2014; Pang et al., 2020; Tang et al., 2011) emphasized that GBE can significantly reduce the level of FBG. Multiple studies (Han et al., 2008; Mao et al., 2008; Wang et al., 2011; Liu et al., 2015) revealed the regulatory effect of GBE on blood lipids, including decreasing serum total cholesterol (TC) and triglyceride (TG) concentrations. AGEs classified as glycotoxins, drive renal pathology via receptor-mediated mechanisms (de Lima et al., 2024). The AGE-RAGE axis activation triggers inflammatory/oxidative cascades and collagen crosslinking, promoting renal tissue destruction, renal fibrosis, vascular sclerosis and other pathological damage (Twarda-Clapa et al., 2022). Chen X. T. et al. (2022) demonstrated that GBE treatment in DN mice significantly downregulated renal cortical expression of RAGE and RhoA proteins, while concurrently elevating levels of arginase-1 and IL-10. This regulatory pattern suggests GBE modulates macrophage polarization toward an anti-inflammatory phenotype via suppression of the AGE/RhoA/RAGE1 signaling axis. Despite these advances, the mechanism of GBE in improving lipid deposition and lipid toxicity in DN remain incompletely characterized and warrants further investigation to elucidate underlying mechanisms.

### Safety of GBE

It is generally believed that GBE has good safety. A meta-analysis (Zhang et al., 2024) showed that there was no statistically significant increase, such as dizziness and rash in the serious adverse reactions of ACEI/ARB combined with GBE. Nevertheless, expanding clinical utilization has revealed emerging safety considerations requiring systematic evaluation. Hu et al.’s systematic review of GBE-associated adverse reactions documented an overall incidence rate of 2.6%, with predominant manifestations including localized pain, abdominal distension, cutaneous erythema, and hypersensitivity responses (Hu et al., 2017). Critically, all reported adverse effects exhibited transient characteristics, resolving completely upon treatment discontinuation or symptomatic management, with no fatalities or chronic sequelae observed. Yao et al. (2025) summarized the date of GBE adverse reactions from the FDA Adverse Event Reporting System (FAERS). The authors concluded that although some adverse events involving different systems have been reported, the quality of the FAERS database is poor, and further clinical trials are needed. Acute and subchronic toxicity studies in SD rats demonstrated that oral administration of GBE at doses ranging from 200 to 4,000 mg/kg for 30 consecutive days did not induce significant toxicity (Shan, 2016). However, one long-term carcinogenicity study reported a significantly increased incidence of hepatocellular carcinoma in mice following daily administration of 2,000 mg/kg GBE for 2 years. It is important to note that this dose substantially exceeds those typically employed in standard pharmacological or toxicity experiments (National Toxicology Program, 2013). In addition, the GBE preparation used in the aforementioned carcinogenicity study contained 10.45 ppm ginkgolic acids, exceeding the limit of ≤5 ppm stipulated by many national pharmacopoeias. It is crucial to recognize that the composition and quality of GBE can vary considerably depending on the extraction and manufacturing processes, leading to potential variability in its efficacy and safety profile (Ude et al., 2013). Therefore, stringent quality control during the production of GBE is essential to ensure product consistency and safety from the source.

### Limitations

This study, while following the PRISMA guidelines, has limitations. (1) The original studies included in this analysis generally provided insufficient chemical characterization of GBE and lacked standardized reporting in accordance with ConPhyMP statement. This deficiency may compromise the reliability and reproducibility of the findings. (2) Evidence of publication bias was detected for some outcome measures, as studies with positive results are more likely to be published. This may lead to an overestimation of the therapeutic efficacy of GBE. (3) A portion of the data was extracted from published figures using digitalization software (WebPlotDigitizer 4.7), which may introduce minor measurement inaccuracies. (4) The number of studies reporting on inflammation and fibrosis biomarkers was limited. This paucity of data may constrain the interpretation of the meta-analysis results regarding these specific mechanistic pathways. (5) Although the random effect model was used for meta-analysis, different measurement methods of outcome indicators may cause systematic errors in the results. (6) The heterogeneity of meta-analysis results cannot be ignored. Although subgroup analysis was performed, the source of heterogeneity was not yet fully clear, which may bring uncertainty to the results. (7) The longest duration of GBE in the study was 3 months, but clinical patients often need longer medication. The efficacy and safety of GBE still need to be further studied to provide more scientific preclinical evidence. (8) Although the protective effect of GBE on the kidney was realized, there are significant differences between animal models and human disease pathology. Therefore, the translational relevance of these preclinical findings to human patients remains to be confirmed in future clinical studies.

## Conclusion

Although studies have introduced the effect of GBE in the treatment of DN, there is no comprehensive analysis of the existing data. We conducted a meta-analysis of preclinical studies on GBE in the treatment of DN, and explored the time-dose-effect relationship of GBE for the first time. The results show that GBE confers protective effects in experimental DN models, mechanistically attributed to inhibition of inflammatory responses, attenuation of oxidative stress, suppression of fibrotic pathways, and modulation of glycolipid metabolism. Based on the importance of dose and duration in administration, it is recommended that the dose of GBE is 36–200 mg/kg/d and the duration is 8–12 weeks in the DN models.

## Data Availability

The original contributions presented in the study are included in the article/Supplementary Material, further inquiries can be directed to the corresponding authors.
