# Supplementary material for: Preclinical evidence of ginkgo biloba extract on diabetic nephropathy: a systematic review and meta-analysis
Source: Front Pharmacol. 2026 Jan 12;16:1717777. doi: 10.3389/fphar.2025.1717777 (PMC12833005; doi:10.3389/fphar.2025.1717777)
Supplement: Supplementary file 1 [file DataSheet1.pdf]

Supplementary Table 1. Search strategy of each database.

Databases	Search strategies
**PubMed**	(("Diabetic Nephropathies"[Mesh]) OR ((((((((((((((((Nephropathies, Diabetic[Title/Abstract]) OR (Nephropathy, Diabetic[Title/Abstract])) OR (Diabetic Kidney Disease[Title/Abstract])) OR (Diabetic Kidney Diseases[Title/Abstract])) OR (Kidney Disease, Diabetic[Title/Abstract])) OR (Kidney Diseases, Diabetic[Title/Abstract])) OR (Diabetic Nephropathy[Title/Abstract])) OR (Diabetic Glomerulosclerosis[Title/Abstract])) OR (Glomerulosclerosis, Diabetic[Title/Abstract])) OR (Intracapillary Glomerulosclerosis[Title/Abstract])) OR (Kimmelstiel-Wilson Disease[Title/Abstract])) OR (Kimmelstiel Wilson Disease[Title/Abstract])) OR (Nodular Glomerulosclerosis[Title/Abstract])) OR (Glomerulosclerosis, Nodular[Title/Abstract])) OR (Kimmelstiel-Wilson Syndrome[Title/Abstract])) OR (Kimmelstiel Wilson Syndrome[Title/Abstract])) OR (Syndrome, Kimmelstiel-Wilson[Title/Abstract]))) AND ((("Ginkgo biloba extract" [Supplementary Concept]) OR (((((((((((Ginkgo leaf extract[Title/Abstract]) OR (Tebokan[Title/Abstract])) OR (Tebonin[Title/Abstract])) OR (EGb 761[Title/Abstract])) OR (EGb-761[Title/Abstract])) OR (EGb761[Title/Abstract])) OR (GBE 761 ONC[Title/Abstract])) OR (Rokan[Title/Abstract])) OR (Tanakan[Title/Abstract])) OR (GBE 761[Title/Abstract])) OR (GBE-761[Title/Abstract])) OR (Ginkgo biloba extract 761[Title/Abstract]))) OR (("Ginkgo biloba"[Mesh]) OR ((((((((((((Gingko[Title/Abstract]) OR (Gingkos[Title/Abstract])) OR (Gingko biloba[Title/Abstract])) OR (Ginkgo[Title/Abstract])) OR (Ginkgos[Title/Abstract])) OR (Ginko[Title/Abstract])) OR (Maidenhair Tree[Title/Abstract])) OR (Maidenhair Trees[Title/Abstract])) OR (Tree, Maidenhair[Title/Abstract])) OR (Trees, Maidenhair[Title/Abstract])) OR (Ginkgophyta[Title/Abstract]))))
**Web of science**	#3 #1 AND #2 and Preprint Citation Index (Exclude – Database)<br>#2 Ginkgo biloba (Topic) or Gingko (Topic) or Gingkos (Topic) or Gingko biloba (Topic) or Ginkgo (Topic) or Ginkgos (Topic) or Ginko (Topic) or Maidenhair Tree (Topic) or Maidenhair Trees (Topic) or Tree, Maidenhair (Topic) or Trees, Maidenhair (Topic) or Ginkgophyta (Topic) or Ginkgo biloba extract (Topic) or Ginkgo leaf extract (Topic) or Tebokan (Topic) or Tebonin (Topic) or EGb 761 (Topic) or EGb-761 (Topic) or EGb761 (Topic) or GBE 761 ONC (Topic) or Rokan (Topic) or Tanakan (Topic) or GBE 761 (Topic) or GBE-761 (Topic) or Ginkgo biloba extract 761 (Topic) and Preprint Citation Index (Exclude – Database)<br>#1 Diabetic Nephropathies (Topic) or Nephropathies, Diabetic (Topic) or Nephropathy, Diabetic (Topic) or Diabetic Kidney Disease (Topic) or Diabetic Kidney Diseases (Topic) or Kidney Disease, Diabetic (Topic) or Kidney Diseases, Diabetic (Topic) or Diabetic Nephropathy (Topic) or Diabetic Glomerulosclerosis (Topic) or Glomerulosclerosis, Diabetic (Topic) or Intracapillary Glomerulosclerosis (Topic) or Kimmelstiel-Wilson Disease (Topic) or Kimmelstiel Wilson Disease (Topic) or Nodular Glomerulosclerosis (Topic) or

	Glomerulosclerosis, Nodular (Topic) or Kimmelstiel-Wilson Syndrome (Topic) or Kimmelstiel Wilson Syndrome (Topic) or Syndrome, Kimmelstiel-Wilson (Topic) and Preprint Citation Index (Exclude – Database)
**Embase**	#8 #3 AND #7<br>#7 #4 OR #5 OR #6<br>#6 'gingko':ab,ti OR 'gingkos':ab,ti OR 'gingko biloba':ab,ti OR 'ginkgo':ab,ti OR 'ginkgos':ab,ti OR 'ginko':ab,ti OR 'maidenhair tree':ab,ti OR 'maidenhair trees':ab,ti OR 'tree, maidenhair':ab,ti OR 'trees, maidenhair':ab,ti OR 'ginkgophyta':ab,ti OR 'ginkgo leaf extract':ab,ti OR 'tebokan':ab,ti OR 'tebonin':ab,ti OR 'egb 761':ab,ti OR 'egb-761':ab,ti OR 'egb761':ab,ti OR 'gbe 761 onc':ab,ti OR 'rokan':ab,ti OR 'tanakan':ab,ti OR 'gbe 761':ab,ti OR 'gbe-761':ab,ti OR 'ginkgo biloba extract 761':ab,ti<br>#5 'ginkgo biloba extract'/exp<br>#4 'ginkgo biloba'/exp<br>#3 #1 OR #2<br>#2 'nephropathies, diabetic':ab,ti OR 'nephropathy, diabetic':ab,ti OR 'diabetic kidney disease':ab,ti OR 'diabetic kidney diseases':ab,ti OR 'kidney disease, diabetic':ab,ti OR 'kidney diseases, diabetic':ab,ti OR 'diabetic glomerulosclerosis':ab,ti OR 'glomerulosclerosis, diabetic':ab,ti OR 'intracapillary glomerulosclerosis':ab,ti OR 'kimmelstiel-wilson disease':ab,ti OR 'kimmelstiel wilson disease':ab,ti OR 'nodular glomerulosclerosis':ab,ti OR 'glomerulosclerosis, nodular':ab,ti OR 'kimmelstiel-wilson syndrome':ab,ti OR 'kimmelstiel wilson syndrome':ab,ti OR 'syndrome, kimmelstiel-wilson':ab,ti<br>#1 'diabetic nephropathy'/exp
**CBM**	7 (#6) AND (#5)<br>6 (#4) OR (#3)<br>5 (#2) OR (#1)<br>4 "白果叶"[常用字段:智能] OR "银杏叶提取物"[常用字段:智能]<br>3 "银杏叶"[不加权:扩展]<br>2 "毛细管间性肾小球硬化症"[常用字段:智能] OR "糖尿病性肾小球硬化症"[常用字段:智能] OR "Kimmelstiel"[常用字段:智能] AND "Wilson 病"[常用字段:智能] OR "结节性肾小球硬化症"[常用字段:智能] OR "糖尿病肾疾病"[常用字段:智能] OR "基-威综合征"[常用字段:智能] OR "Kimmelstiel-Wilson 病"[常用字段:智能] OR "糖尿病肾脏病"[常用字段:智能] OR "糖尿病肾脏疾病"[常用字段:智能] OR "糖尿病性肾病"[常用字段:智能] OR "糖尿病性肾小球硬化"[常用字段:智能]<br>1 "糖尿病肾病"[不加权:扩展]

**CNKI**	SU%=('糖尿病肾病' + '糖尿病肾脏病' + '糖尿病肾脏疾病' + '糖尿病性肾病' + '糖尿病性肾小球硬化') * ('银杏叶提取物' + '银杏叶')
**WanFang**	主题: (("糖尿病肾病" or "糖尿病肾脏病" or "糖尿病肾脏疾病" or "糖尿病性肾病" or "糖尿病性肾小球硬化") and ("银杏叶提取物" or "银杏叶"))
**VIP**	M=((糖尿病肾病 OR 糖尿病肾脏病 OR 糖尿病肾脏疾病 OR 糖尿病性肾病 OR 糖尿病性肾小球硬化) AND (银杏叶提取物 OR 银杏叶))

Supplementary Table 2. Risk bias evaluations.

序号	研究	Sequence generation	Baseline characteristics	Allocation concealment	Random housing	Caregiver blinding	Random outcome assessment	Outcome assessor blinding	Incomplete outcome data	Selective outcome reporting	Other sources of bias
1	Chang 2021	U	U	U	L	H	U	U	U	L	L
2	Chen 2006a	U	U	U	L	H	U	U	L	L	L
3	Chen 2006b	U	U	U	L	H	U	U	L	L	L
4	Chen 2024	U	L	U	L	H	U	U	L	L	L
5	Du 2019	U	U	U	L	H	U	U	L	L	L
6	Han 2008	U	U	U	L	H	U	U	L	L	L
7	Hou 2005	U	U	U	L	H	U	U	L	L	L
8	Jiang 2022	U	U	U	L	H	U	U	L	L	L
9	Jing 2005	U	U	U	L	H	U	U	L	L	L
10	Li 2011	U	L	U	L	H	U	U	L	L	L
11	Li 2019	L	L	U	L	H	U	U	L	L	L
12	Liao 2000	U	U	U	U	H	U	U	L	L	L
13	Liu 2008	U	U	U	L	H	U	U	U	L	L
14	Liu 2015	U	U	U	L	H	U	U	L	L	L
15	Liu 2024	U	U	U	U	H	U	U	L	L	L
16	Lu 2007	U	L	U	L	H	U	U	L	L	L
17	Lu 2015	U	L	U	L	H	U	U	L	L	L
18	Mao 2008	U	U	U	L	H	U	U	L	L	L
19	Mu 2014	U	U	U	L	H	U	U	U	L	L
20	Nian 2003	U	L	U	L	H	U	U	L	L	L

21	Pang 2020	U	U	U	L	H	U	U	L	L	L
22	Su 2003	U	U	U	L	H	U	U	L	L	L
23	Tang 2011	U	U	U	U	H	U	U	L	L	L
24	Wang 2011	U	U	U	L	H	U	U	L	L	L
25	Yang 2011	U	U	U	L	H	U	U	L	L	L
26	Yin 2003	U	U	U	L	H	U	U	L	L	L
27	Zhang 2008	U	L	U	L	H	U	U	L	L	L
28	Zhang 2017	U	L	U	L	H	U	U	L	L	L
29	Zhang 2018	U	L	U	L	H	U	U	L	L	L
30	Zheng 2011	U	U	U	L	H	U	U	L	L	L

Supplementary Table 3. Relevant information on the GBE.

Author/Year	Manufacturer	Batch number
Chang 2021	Dr. Willmar Schwabe, GmbH & CO.KG	2430819
Chen 2006a	Beijing Double-Crane Natural Pharmaceuticals Co., Ltd.	20404.0101
Chen 2006b	Beijing Double-Crane Natural Pharmaceuticals Co., Ltd.	319163.0405
Chen 2024	Dr. Willmar Schwabe, GmbH & CO.KG	4380420
Du 2019	Jiangsu Nhwa Pharmaceutical Co., Ltd.	NR
Han 2008	Beijing Double-Crane Natural Pharmaceuticals Co., Ltd.	20404.0101
Hou 2005	Jiangsu Haici Pharmaceutical Co., Ltd.	NR
Jiang 2022	Hainan Qili Pharmaceutical Co., Ltd.	Z20053069
Jing 2005	Zhejiang Conba Pharmaceutical Co., Ltd.	Z20027963
Li 2011	Shanghai Sine Bairui Da Pharmaceutical Co., Ltd.	691004
Li 2019	Zhejiang Conba Pharmaceutical Co., Ltd.	Z20027963
Liao 2000	Shanghai Green Valley Source Industrial Co., Ltd.	980705
Liu 2008	Pizhou Fuwei Biochemical Co., Ltd.	51023
Liu 2015	Zhejiang Conba Pharmaceutical Co., Ltd.	NR
Liu 2024	Jiangsu Yangtze River Pharmaceutical Group Co., Ltd.	Z20027949
Lu 2007	Pizhou Fuwei Biochemical Co., Ltd.	51023
Lu 2015	Huike Botanical Development Co., Ltd	HK20121201
Mao 2008	Jiangsu Yangtze River Pharmaceutical Group Co., Ltd.	NR
Mu 2014	Guilin Site New Technology Co., Ltd.	NR
Nian 2003	Hunan Lushan Natural Plant Pharmaceutical Co., Ltd.	NR
Pang 2020	Dr. Willmar Schwabe, GmbH & CO.KG	2280219
Su 2003	Xuzhou Huakang Biological Products Co., Ltd.	NR
Tang 2011	NR	NR
Wang 2011	Guilin Site New Technology Co., Ltd.	NR
Yang 2011	NR	NR
Yin 2003	Department of Medicinal Chemistry, Xuzhou Medical University	20020415
Zhang 2008	Beijing Double-Crane Natural Pharmaceuticals Co., Ltd.	NR
Zhang 2017	Zhejiang Conba Pharmaceutical Co., Ltd.	Z20027963
Zhang 2018	Zhejiang Conba Pharmaceutical Co., Ltd.	Z20027963
Zheng 2011	Jisheng Chemical & Pharmaceutical Co., Ltd., Taiwan, China	NR

Supplementary Table 4. Subgroup analysis of primary outcome indicators.

Variables	SMD [95%CI]	*P* value	I^2(%)	*P*-heterogeneity
**FBG**				
DN models				
type 1 DN	-1.25 [-1.65, -0.85]	0.000	72.3	0.000
type 2 DN	-1.41 [-2.09, -0.73]	0.000	54.4	0.052
administration method				
oral gavage	-1.09 [-1.41, -0.77]	0.000	56.7	0.001
intraperitoneal injection	-2.13 [-3.31, -0.96]	0.000	83.2	0.000
duration				
<8 weeks	-1.37 [-2.38, -0.37]	0.007	82.6	0.000
8≤t < 12 weeks	-1.13 [-1.63, -0.63]	0.000	60.3	0.005
≥12 weeks	-1.38 [-1.92, -0.84]	0.000	68.4	0.001
species				
rats	-1.28 [-1.65, -0.90]	0.000	71.2	0.000
mice	-1.35 [-2.21, -0.50]	0.002	44.6	0.165
**SCr**				
DN models				
type 1 DN	-1.41 [-1.74, -1.07]	0.000	31.9	0.136
type 2 DN	-2.06 [-2.68, -1.44]	0.000	22.2	0.278
administration method				
oral gavage	-1.59 [-1.94, -1.24]	0.000	42.4	0.047
intraperitoneal injection	-1.31 [-2.09, -0.53]	0.001	30.6	0.230
duration				
<8 weeks	-0.91 [-1.84, -0.01]	0.054	—	—
8≤t < 12 weeks	-1.55 [-2.23, -0.86]	0.000	61.8	0.023
≥12 weeks	-1.65 [-1.99, -1.32]	0.000	10.9	0.344
species				
rats	-1.50 [-1.81, -1.18]	0.000	34.7	0.097
mice	-2.17 [-3.78, -0.56]	0.008	70.5	0.066
**BUN**				
DN models				
type 1 DN	-1.36 [-1.72, -1.00]	0.000	51.4	0.016
type 2 DN	-1.63 [-2.41, -0.84]	0.000	59.4	0.060
administration method				
oral gavage	-1.32 [-1.63, -1.01]	0.000	38.2	0.066

intraperitoneal injection	-1.98 [-3.48, -0.47]	0.010	83.4	0.014
duration				
<8 weeks	-2.22 [-3.29, -1.15]	0.000	62.7	0.101
8≤t < 12 weeks	-1.10 [-1.60, -0.60]	0.000	50.6	0.048
≥12 weeks	-1.48 [-1.80, -1.16]	0.000	0.0	0.603
species				
rats	-1.35 [-1.68, -1.02]	0.000	49.3	0.016
mice	-2.10 [-3.67, -0.52]	0.009	70.1	0.068
**24h Upro**				
DN models				
type 1 DN	-1.41 [-1.97, -0.86]	0.000	70.1	0.000
type 2 DN	-1.53 [-2.33, -0.73]	0.000	38.2	0.198
administration method				
oral gavage	-1.56 [-2.00, -1.13]	0.000	47.4	0.040
intraperitoneal injection	-0.93 [-2.37, -0.51]	0.205	85.6	0.001
duration				
<12 weeks	-0.48 [-2.46, -1.49]	0.630	84.6	0.011
8≤t < 12 weeks	-1.74 [-2.31, -1.17]	0.000	40.8	0.133
≥12 weeks	-1.43 [-1.98, -0.87]	0.000	49.8	0.076
species				
rats	-1.37 [-1.85, -0.90]	0.000	65.2	0.001
mice	-2.26 [-3.41, -1.12]	0.000	—	—
**KI**				
DN models				
type 1 DN	-1.94 [-2.56, -1.33]	0.000	74.4	0.000
type 2 DN	-2.20 [-3.86, -0.54]	0.009	85.7	0.000
administration method				
oral gavage	-1.98 [-2.66, -1.30]	0.000	78.2	0.000
intraperitoneal injection	-1.96 [-3.07, -0.86]	0.001	74.1	0.021
duration				
<12 weeks	-1.93 [-3.17, -0.70]	0.002	83.8	0.000
8≤t < 12 weeks	-1.89 [-2.72, -1.07]	0.000	74.2	0.000
≥12 weeks	-2.32 [-3.80, -0.84]	0.002	84.4	0.002
species				
rats	-1.91 [-2.47, -1.35]	0.000	72.0	0.000
mice	-2.66 [-5.21, -0.11]	0.041	90.3	0.000

DN: diabetic nephropathy; FBG: fasting blood glucose; BUN: blood urea nitrogen; SCr: serum creatinine; 24h Upro: 24-hour urine protein; KI: kidney index; SMD: standardized mean differences

Supplementary Table 5. Results from trim-and-fill analysis.

Variables	No. of Trials	SMD [95%CI]	*P* value
**FBG**			
before trim and fill	27	-1.28 [-1.63, -0.94]	0.000
after trim and fill	29	-1.39 [-1.74, -1.04]	0.000
**KI**			
before trim and fill	16	-1.95 [-2.53, -1.38]	0.000
after trim and fill	17	-2.10 [-2.69, -1.50]	0.000

FBG: fasting blood glucose; KI: kidney index; SMD: standardized mean differences

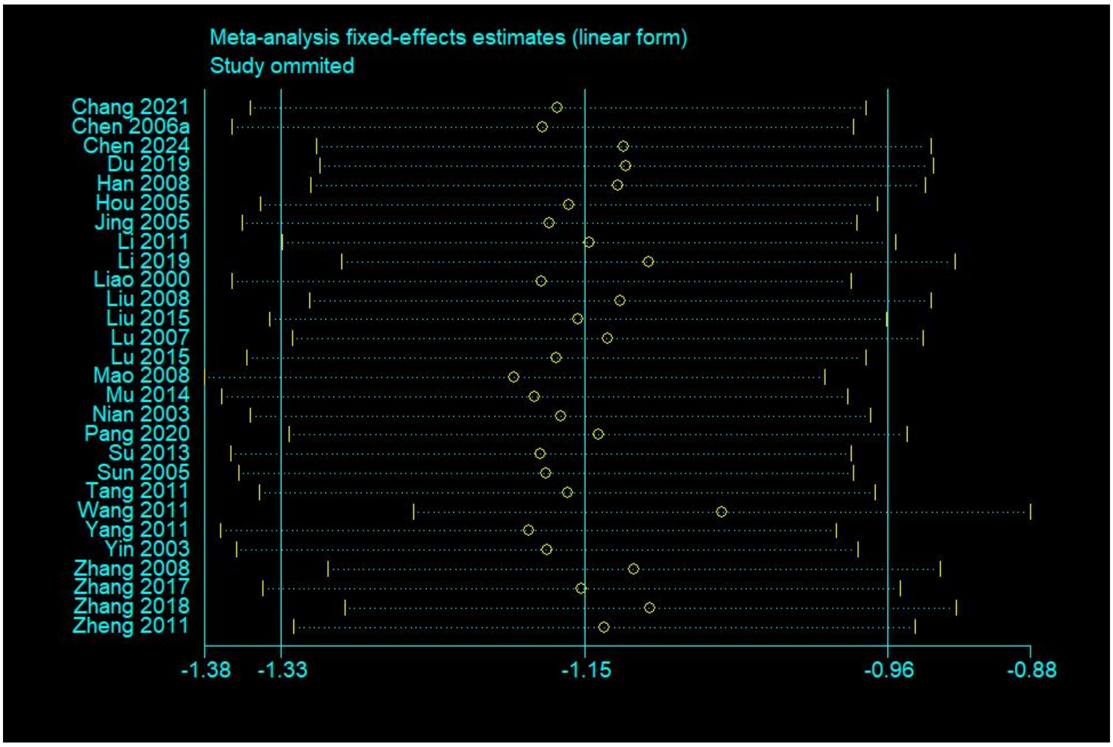

Sensitivity analysis of FBG

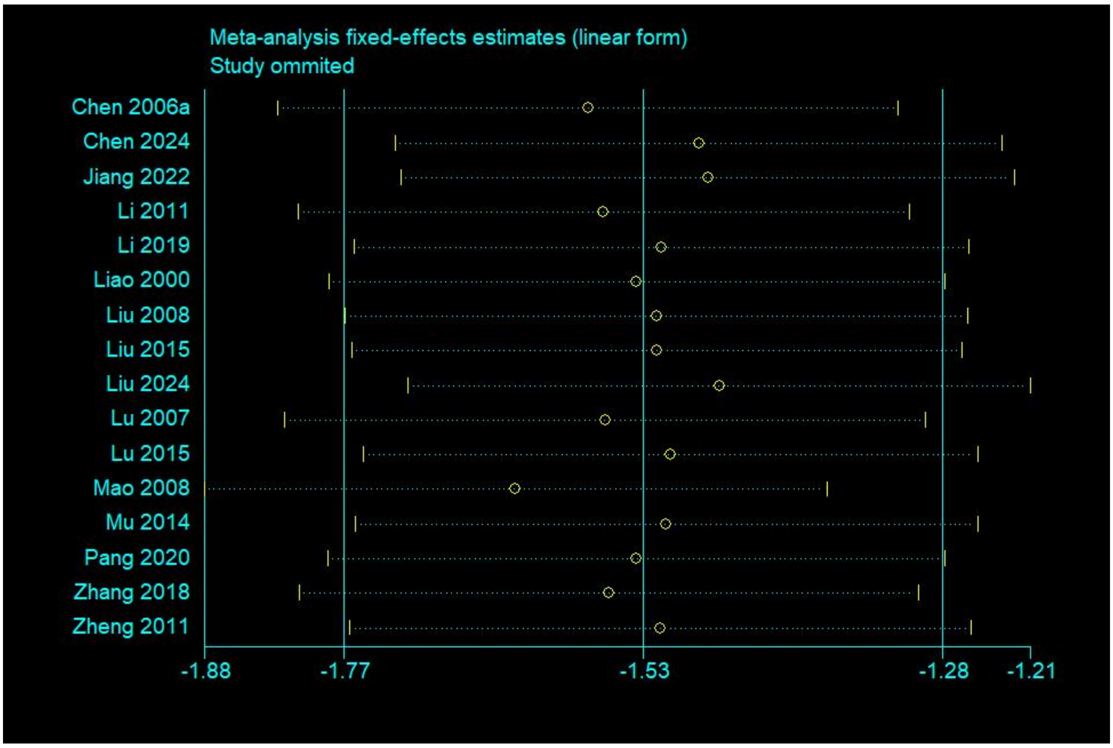

Sensitivity analysis of SCr

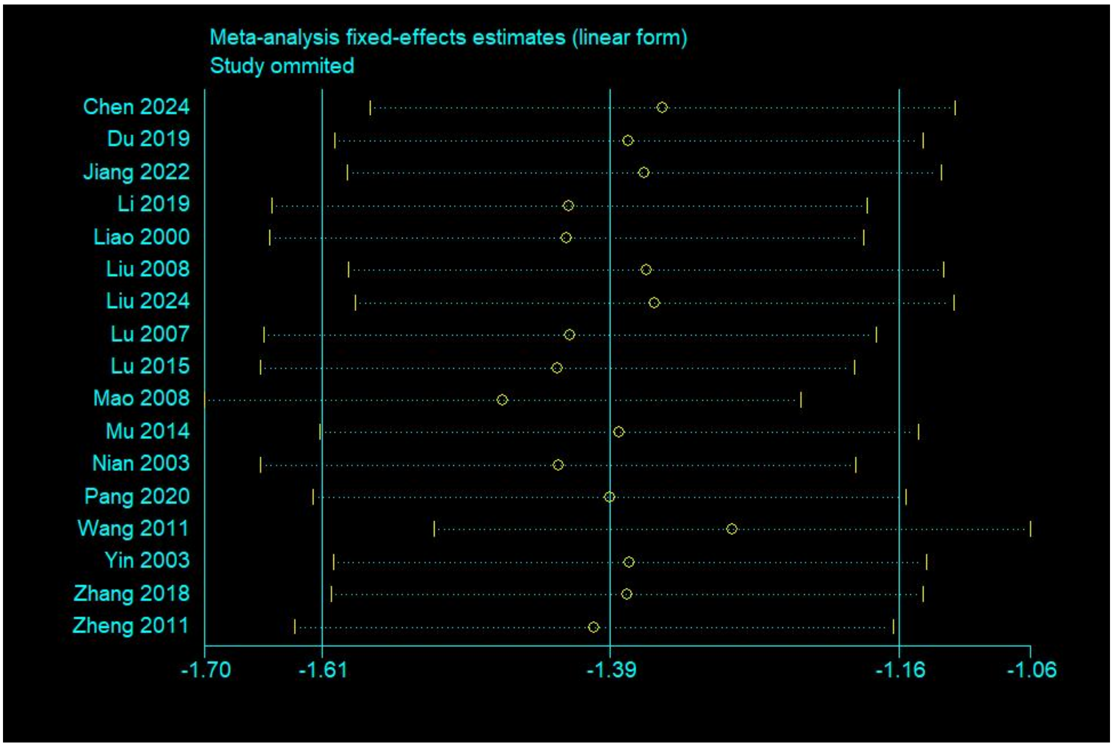

Sensitivity analysis of BUN

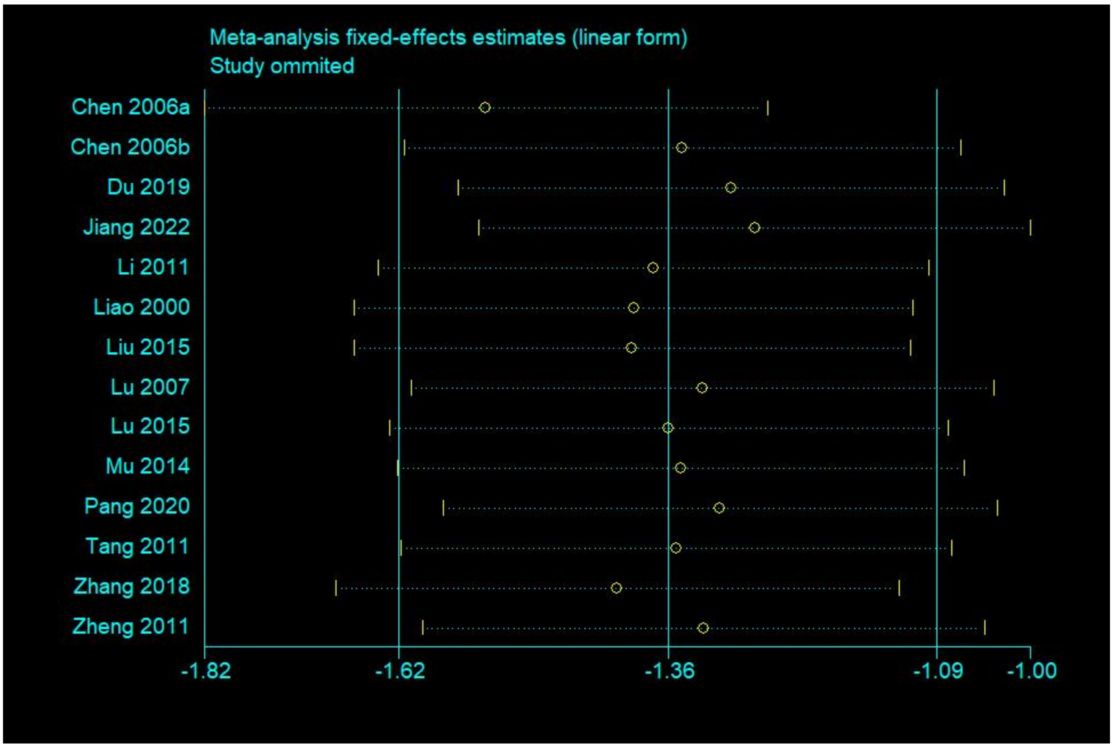

Sensitivity analysis of 24 h UPro

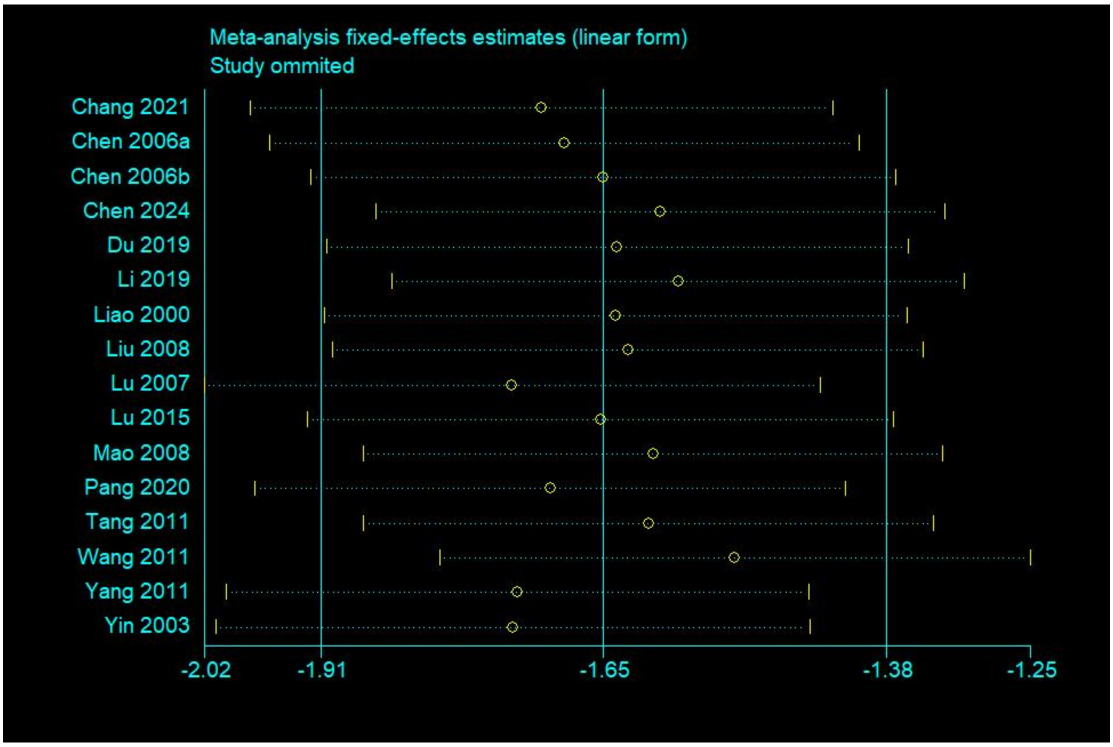

Sensitivity analysis of KI

Supplementary Figure 1. Sensitivity analysis

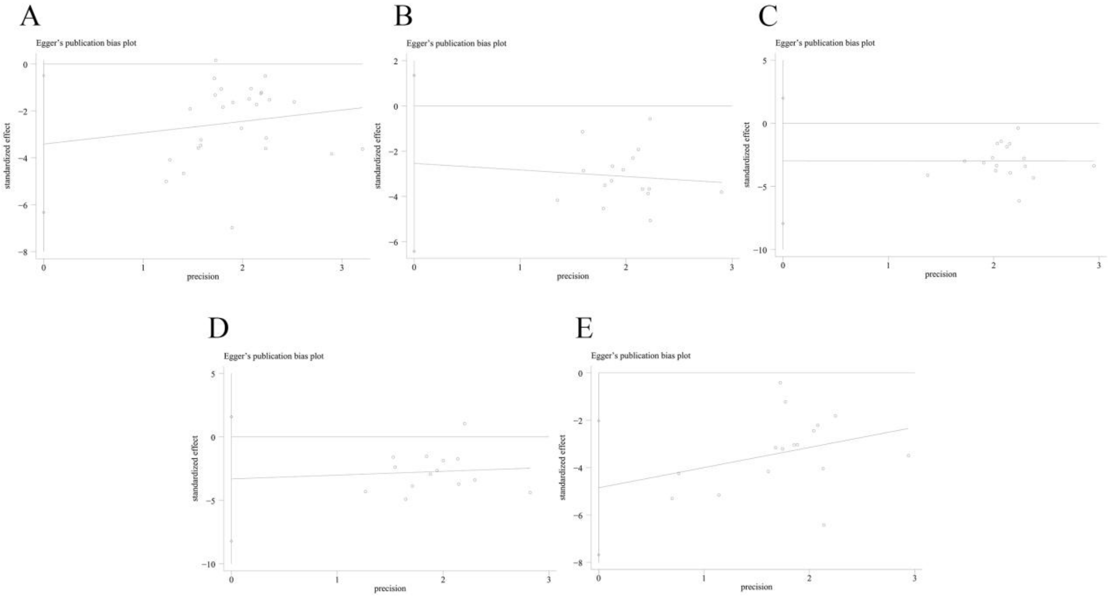

Supplementary Figure 2. Egger's publication bias plot for (A) FBG, (B) SCr, (C) BUN, (D) 24h Upro, (E) KI.

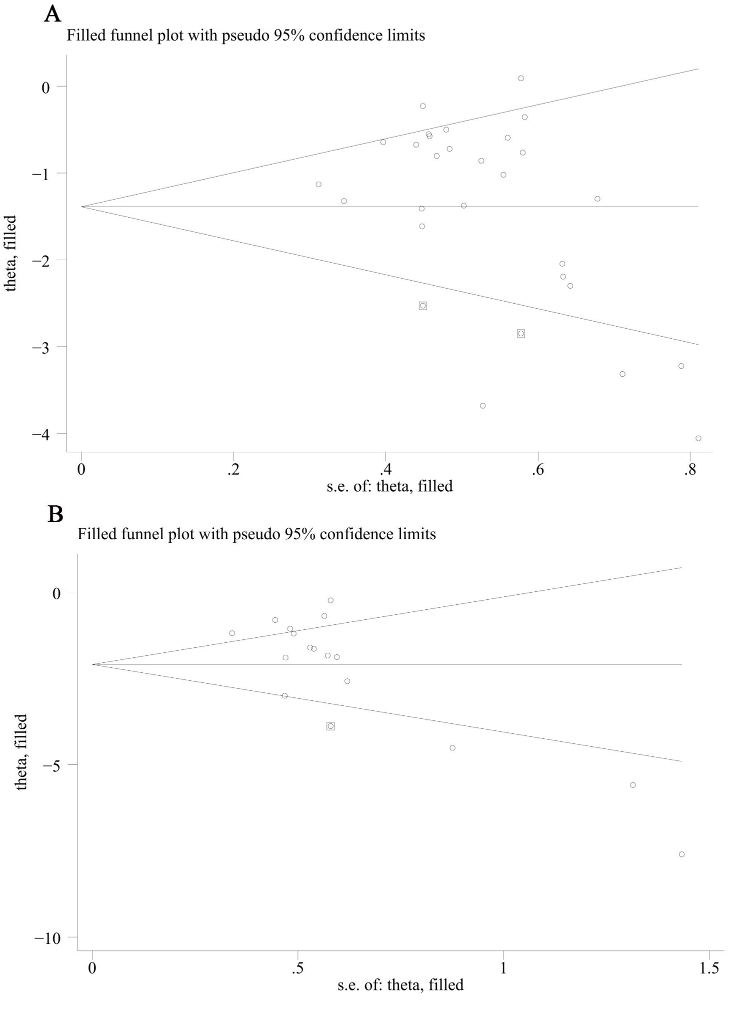

Supplementary Figure 3. Trim-and-fill analysis for (A) FBG and (B) KI.

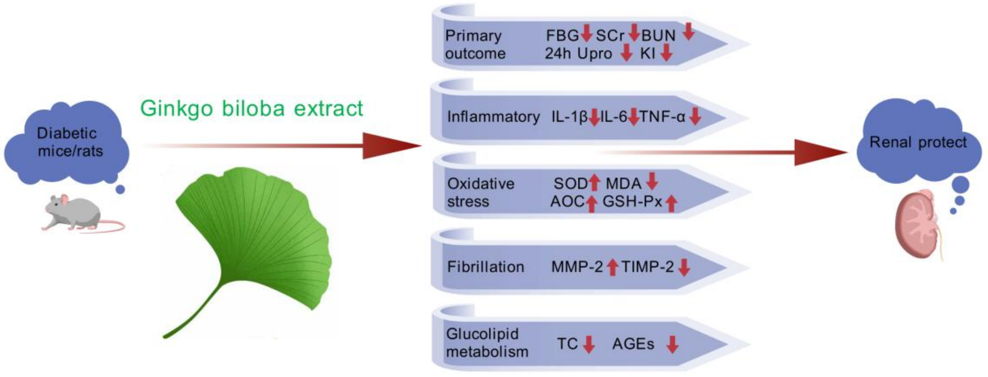

Graphical abstract
